# Loss of *Mst1/2* activity promotes non-mitotic hair cell generation in the neonatal organ of Corti

**DOI:** 10.1038/s41536-022-00261-4

**Published:** 2022-10-25

**Authors:** Xiaoling Lu, Huiqian Yu, Jiaoyao Ma, Kunkun Wang, Luo Guo, Yanping Zhang, Boan Li, Zehang Zhao, Huawei Li, Shan Sun

**Affiliations:** 1 ENT Institute and Department of Otorhinolaryngology, Eye & ENT Hospital, State Key Laboratory of Medical Neurobiology and MOE Frontiers Center for Brain Science, NHC Key Laboratory of Hearing Medicine (Fudan University), Fudan University, 200031 Shanghai, P. R. China; 2 Xiamen University School of Life Sciences, 361100 Xiamen, P. R. China; 3 Institutes of Biomedical Sciences, Fudan University, 200032 Shanghai, P. R. China; 4 The Institutes of Brain Science and the Collaborative Innovation Center for Brain Science, Fudan University, 200032 Shanghai, China

**Keywords:** Translational research, Mechanisms of disease, Regeneration

## Abstract

Mammalian sensory hair cells (HCs) have limited capacity for regeneration, which leads to permanent hearing loss after HC death. Here, we used in vitro RNA-sequencing to show that the Hippo signaling pathway is involved in HC damage and self-repair processes. Turning off Hippo signaling through *Mst1/2* inhibition or *Yap* overexpression induces YAP nuclear accumulation, especially in supporting cells, which induces supernumerary HC production and HC regeneration after injury. Mechanistically, these effects of Hippo signaling work synergistically with the Notch pathway. Importantly, the supernumerary HCs not only express HC markers, but also have cilia structures that are able to form neural connections to auditory regions in vivo. Taken together, regulating Hippo suggests new strategies for promoting cochlear supporting cell proliferation, HC regeneration, and reconnection with neurons in mammals.

## Introduction

Hair cell (HC) damage causes permanent sensorineural hearing loss and affects millions of people all over the world^1,2^. Damaged adult mammalian HCs lack regenerative ability^3,4^, although supporting cells (SCs) are a potential resource for regenerating HCs^5,6^. When HCs are damaged in the neonatal mammalian cochlea, SCs can regenerate HCs either through mitotic regeneration or through direct trans-differentiation after activating stem cells or progenitor cells, but this regenerative capacity is quite limited^7–11^. Importantly, HC regeneration does not occur when ablation of HCs is induced after one week of age in vivo^12,13^. The interactions and mechanisms underlying regeneration require further research, particularly in the context of cellular damage paradigms.

Hippo signaling is a highly conserved signaling pathway that plays pleiotropic roles in modulating cell proliferation, differentiation, survival, and death^14^. In mammals, the Hippo pathway consists of the serine/threonine kinases mammalian sterile 20-like kinase 1/2 (MST1/2) and large tumor suppressor 1/2 (LATS1/2), while Yes-associated protein (YAP) is the major downstream mediator of the Hippo pathway^15–17^. Upon stimulation, phosphorylated MST1/2-SAV1 activates the LATS1/2-MOB1a/b complex, which in turn phosphorylates the downstream transcriptional effector YAP to promote its cytoplasmic localization, which is called Hippo-on^18^. In the absence of phosphorylation, YAP is diverted into the nucleus (Hippo-off), where it cooperates with the transcriptional cofactor *Tead* to regulate its target genes that are involved in cell proliferation and differentiation^19,20^.

Previous studies focused on the important role of Hippo signaling in tumorigenesis because the upregulation of Hippo signaling promotes cell growth and inhibits cell apoptosis in almost all tumor tissues^21^, especially its cross-talk with other signaling pathways^22,23^. Comparative microarray analysis demonstrated that *Yap* deficiency-induced gene expression changes in the Wnt signaling pathway, and this may be associated with HC (neuromast) regeneration in zebrafish^24^. In the auditory system, Gnedeva et al. reported that the conditional loss of *Yap* was associated with cell cycle exit during the embryonic stage in the organ of Corti and led to significantly smaller sensory organs^25^. The findings by Rudolf et al. suggested that cellular damage overcomes inhibitory Hippo signaling and facilitates regenerative proliferation in non-mammalian utricles, whereas suppressing YAP-TEAD signaling in mammalian utricles contributes to maintaining the proliferative quiescence^26^. The different cellular localizations of YAP may partially account for the regenerative abilities of the utricles in chicks^27^. However, most studies on the Hippo signaling pathway have focused on its function in inhibiting cell proliferation and promoting withdrawal from the cell cycle in the inner ear, and not on its application for promoting HC regeneration.

In this study, we focused on Hippo signaling in the mouse cochlea and found that it is involved in the processes of HC regeneration. We further found that YAP nuclear accumulation is increased after neomycin insult, and promoting YAP to enter the nucleus results in increased supernumerary HC generation in neonatal mice. More importantly, our results demonstrated that the newly generated HCs induced after *Mst1/2* knockout in Sox9-positive supporting cells (SCs) were functional in vivo, which is particularly noteworthy in mammals.

## Results

### Hippo signaling was involved in the regeneration process in damaged cochleae in vitro

**Fig. 1:**
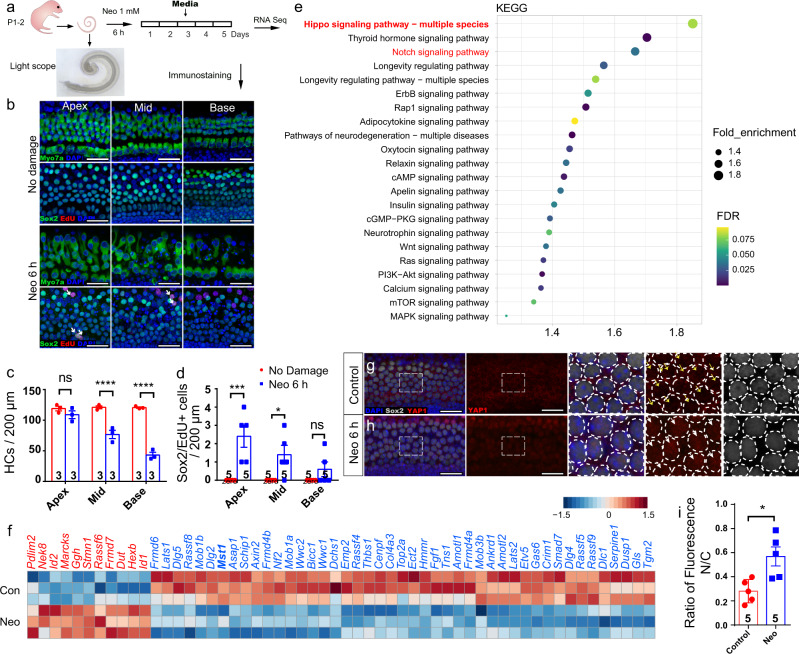
Hippo signaling is involved during post-injury and self-repair processes in damaged neonatal mammalian cochleae in vitro. **a** Cochleae of P2 neonatal mice were isolated and cultured for 12 h, then damaged with 1 mM neomycin for 6 h and cultured for another 5 days with 10 μM EdU. **b**, **c** After neomycin damage, HC death increased from the apex to the base of the cochlea (Myosin7a+ HCs, apex: 109.0 ± 6.4; middle: 76.7 ± 6.7; base: 43.0 ± 4.6), and two-way ANOVA followed by Sidak’s multiple comparison test showed that there was a significant difference in the middle and basal turns. **d** Very few proliferative SCs were observed after HC damage (Sox2/EdU double-positive SCs, apex: 2.4 ± 0.6; middle: 1.4 ± 0.5; base: 0.6 ± 0.4), although two-way ANOVA followed by Sidak’s multiple comparison test showed the significant difference in the apical and middle turns. **e** Kyoto Encyclopedia of Genes and Genomes (KEGG) pathway analysis of the transcriptome data. **f** Heatmap showing relative expression levels of the differentially expressed genes associated with the Hippo signaling pathway after HC damage. (FDR < 0.01; *n* = 3 for each condition). Higher expressed genes are shown in red, while the genes with relatively lower levels are depicted in blue. **g**–**i** Cochleae of P2 mice were dissected and cultured for 12 h and then treated with 1 mM neomycin for 6 h. Immunohistochemical staining was conducted after another 24 h culture. Compared with control groups, immunohistochemical staining and relative fluorescence quantitative analysis results analyzed with two-tailed, unpaired Student’s *t* tests showed that YAP (red) nuclear accumulation in Sox2+ SCs (white) was increased after neomycin injury. *p < 0.05, **p < 0.01, ***p < 0.001, ****p < 0.0001. Data are shown as the mean ± SEM. Scale bars = 20 μm.

Aminoglycoside antibiotics (such as neomycin) are known toxins to cochlear HCs, and progressive HC loss was found in cochleae from the apical to basal turns after neomycin treatment (Fig. 1a–c, Fig. S1). Meanwhile, very few proliferative SCs were observed in such situations, and these were mostly distributed in the apical turns (Fig. 1d). In order to explore the molecular mechanism based on SC proliferation, principal component analysis (PCA) of normalized RNA-seq data was performed to investigate overall variances between and within the two groups. Neomycin-treated cells and control cells formed distinct clusters along PC1, which explained 86% of the variance. Within-group variance was revealed by PC2, which accounted for only 7% of the variance. From the volcano plot and Gene Ontology analysis, we found that the ribonucleoprotein complex and ribosome biogenesis were significantly upregulated, which was closely related to cell proliferation or regeneration (Fig. S2). Kyoto Encyclopedia of Genes and Genomes pathway analysis showed that large numbers of signaling pathways, including Hippo and Notch, were involved in the process of HC death or SC proliferation (Fig. 1e). We focused on changes in Hippo signaling after HC damage and found that 56 genes in the Hippo signaling pathway and among its downstream genes were associated with HC damage and repair processes, including *Mst1, Lats1/2*, *Mob1b*, and *Amotl2* (Fig. 1f).

We further observed that YAP nuclear accumulation in SCs could be triggered by neomycin insult (Fig. 1g–i). It has been reported that YAP nuclear accumulation rapidly decreases in the organ of Corti from embryos to newborns^25^, as confirmed in our results (Fig. S3), which suggests that YAP translocation from the cytoplasm to the nucleus is associated with the regenerative capacity of cochlear cells.

### Hippo-off promoted SC proliferation and led to supernumerary HCs

**Fig. 2:**
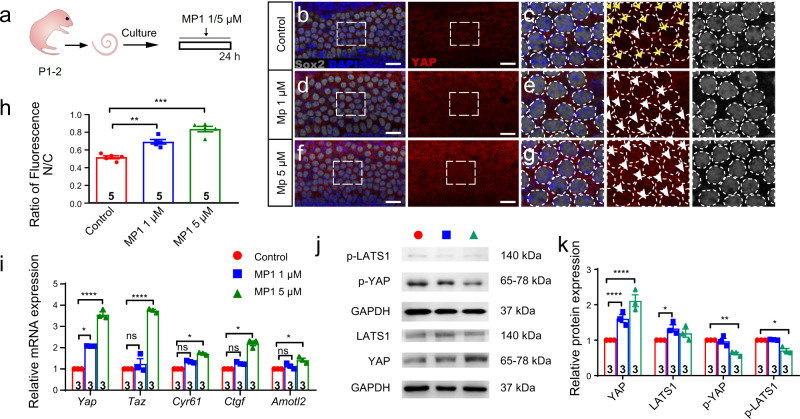
Turning off Hippo signaling induces YAP nuclear accumulation and subsequent interaction with its target genes. **a** Cochleae of P2 neonatal mice were isolated and cultured. After 12 h, 1 μM or 5 μM XMU-MP-1 was added for 24 h. **b**–**g** The distribution of YAP (red) in Sox2+ SCs (white) from P2 neonatal control and 1 µM or 5 μM XMU-MP-1-treated mouse cochleae, respectively. The yellow arrows indicate that the YAP nuclear accumulation was very low in the control group, while the white arrows indicate that the YAP nuclear accumulation was increased in the XMU-MP-1 groups. **h** The relative florescence of YAP in the nuclei and cytoplasm of SCs in the control and XMU-MP-1-treated groups was analyzed with one-way ANOVA followed by Dunnett’s multiple comparisons test, indicating that XMU-MP-1 significantly promoted the translocation of YAP into the nucleus. **i** The relative mRNA expression levels of Hippo pathway-related genes and downstream genes after exogenous XMU-MP-1 treatment analyzed with one-way ANOVA followed by Tukey’s multiple comparisons test. **j**, **k** The western blots and the relative fluorescence quantification of p-YAP, p-LATS, YAP, and LATS in the control group and the XMU-MP-1 groups analyzed with two-way ANOVA followed by Dunnett’s multiple comparisons test. *p < 0.05, **p < 0.01, ***p < 0.001, ****p < 0.0001. Data are shown as the mean ± SEM. Scale bars = 20 μm.

XMU-MP-1, a selective MST1/2 inhibitor^28^, was used to pharmacologically turn-off Hippo signaling in the mouse cochlea (Fig. 2a). YAP was increased in the SC nucleus (Fig. 2b–h), and the expression of Hippo-related downstream genes was increased in a dose-dependent manner (Fig. 2i). The ratio of nuclear YAP was increased and the level of phosphorylated YAP was decreased in the organ of Corti following XMU-MP-1 incubation, while the level of phosphorylated LATS-1 was decreased (Figs. 2j, k, S4).

**Fig. 3:**
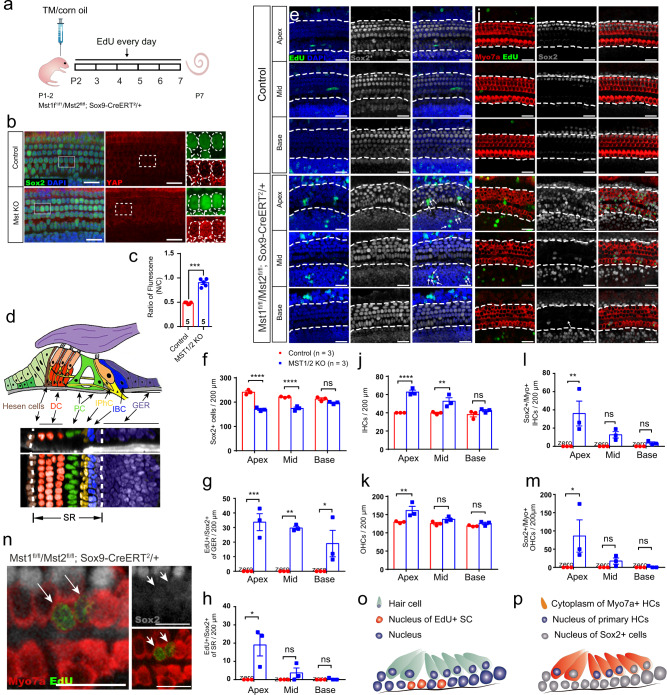
Turning off Hippo signaling initiates the proliferation of SCs and the direct differentiation of HCs from SCs in vivo. **a** Mst1^f1/f1^/Mst2^fl/fl^; Sox9-CreERT^2^/+ mice were used to knock out *Mst1/2* in Sox9+ SCs and thus turn-off Hippo signaling to promote YAP nuclear translocation. Tamoxifen was injected at P1–2, and EdU was injected from P2–7 and the cochleae were harvested at P7. **b** Compared with the control groups, there was much more YAP nuclear translocation in Mst1^f1/f1^/Mst2^fl/fl^; Sox9-CreERT^2^/+ mice. The nuclei are circled in white. Compared with controls, more YAP was transferred into the cell nucleus in the *Mst1/2* knockout group. **c** The relative florescence of YAP in the nuclei and cytoplasm of SCs in control and *Mst1/2* knockout mice was analyzed with two-tailed, unpaired Student’s *t* tests, indicating that *Mst1/2* knockout promoted the translocation of YAP into the nucleus. **d** The schematic diagram of the sectional views of the organ of Corti. DC, Deiters’ cell; PC, pillar cell; IPhC, inner phalangeal cell; IBC, inner border cell; GER, greater epithelial ridge. **e**–**h** Very few EdU+ SCs were detected in control mice, and two-way ANOVA followed by Sidak’s multiple comparison test showed that there were significantly greater numbers of Sox2+/EdU+ SCs in the SR and GER with the total amount of Sox2+ SCs decreasing in Mst1^f1/f1^/Mst2^fl/fl^; Sox9-CreERT^2^/+ mice. **i**–**m** There were large numbers of newly generated HCs among both the IHCs and OHCs, and many Sox2+/Myosin7a+ HCs were detected in both the IHCs and OHCs of Mst1^f1/f1^/Mst2^fl/fl^; Sox9-CreERT^2^/+ mice (**i**), and two-way ANOVA followed by Sidak’s multiple comparison test was performed to show the significant differences. **n** Very few Myosin7a+/EdU+ HCs, about 0–2 per cochlea, were observed in Mst1^f1/f1^/Mst2^fl/fl^; Sox9-CreERT^2^/+ mice. **o** The schematic diagram of SC proliferation in *Mst1/2* knockout mice in vivo. **p** The schematic diagram of supernumerary HCs in *Mst1/2* knockout mice in vivo. *p < 0.05, **p < 0.01, ***p < 0.001, ****p < 0.0001. Data are shown as the mean ± SEM. Scale bars = 20 μm.

We next generated Mst1^fl/fl^/Mst2^fl/fl^; Sox9-CreERT^2^/+ transgenic mice to turn-off Hippo specifically in SCs in vivo (Fig. 3a), which confirmed that conditional knockout of *Mst1/2* in SCs drives YAP translocation into the SC nuclei (Fig. 3b, c). The schematic diagram in Fig. 3d shows the structures and cell subtypes of the cochlea. We confirmed that there was no significant difference between Mst1^fl/fl^/Mst2^fl/fl^ transgenic mice and wild-type (WT) mice without Cre activation (Fig. S5). With tamoxifen injection in the WT control groups, no EdU+/Sox2+ cells were observed at postnatal day (P)7, indicating that postnatal SCs were mitotically quiescent after birth (Fig. 3e). However, tamoxifen injection resulted in a significant number of Sox2+/EdU+ cells in the *Mst1/2* knockout mice in both the sensory region (SR) and the greater epithelial ridge (GER) (Fig. 3e–h). Specifically, several Hippo signaling-related genes were changed at both the transcription and translation levels (Figs. S6, S7).

Myosin7a was used as the marker for HCs, while Sox2 was used as the SC marker^29,30^. In the HC layer in P7 mice, there were large numbers of Myosin7a+/Sox2+ cells in the transgenic mice, but none were observed in control littermates. The total numbers of both inner HCs (IHCs) and outer HCs (OHCs) were significantly greater, while the total number of Sox2+ cells in the SC layer was smaller in the transgenic mice than in controls (Fig. 3i, j, k). More interestingly, there were significantly greater numbers of Myosin7a+/Sox2+ cells in the transgenic mice, with a decreasing trend from the apex to the base in the cochlea (Fig. 3l, m). It has been reported that some cells that are Myosin7a+ also have Sox2+ nuclei, suggesting that they are newly generated HCs from Sox2+ cells but still at an immature stage^30,31^. Thus, the direct differentiation of SCs into HCs via YAP nuclear translocation resulted in the increased number of HCs and the decreased number of SCs (Fig. 3f, j, k). Furthermore, rare Myosin7a+/EdU+ cells were found in the transgenic mice, which indicated the existence of some mitotic HC generation (Fig. 3n). In conclusion, both direct differentiation and mitotic generation of HCs from SCs via YAP nuclear translocation occurred simultaneously in *Mst1/2* conditional knockout mice, but direct differentiation was the dominant mechanism (Fig. 3o, p).

Sox9 is a member of the Sox family of transcriptional regulators. It is expressed mainly in SCs in the developmental stage of the inner ear^32^. To identify the origin of the proliferating SCs and supernumerary HCs induced by YAP nuclear translocation, the Mst1^fl/fl^/Mst2^fl/fl^; Sox9-CreERT^2^/+; Rosa26-tdTomato/+ mouse lines were created to conditionally knockout *Mst1* and *Mst2* in the Sox9-tdTomato+ SCs. The results suggested that both the proliferative SCs and the differentiated HCs originated from Sox9+ SCs. In addition, the infrequent Myosin7a+/EdU+ cells were also derived from Sox9-tdTomato+ cells and corresponded to mitotically generated HCs in the lineage-traced Sox9+ SCs (Fig. S8). In summary, most of the supernumerary HCs were Sox9-tdTomato+/EdU–, again illustrating that direct differentiation dominated HC generation upon inhibiting Hippo signaling and that these new HCs derived from Sox9-tdTomato+ SCs.

### The supernumerary HCs were functional but immature in vivo

**Fig. 4:**
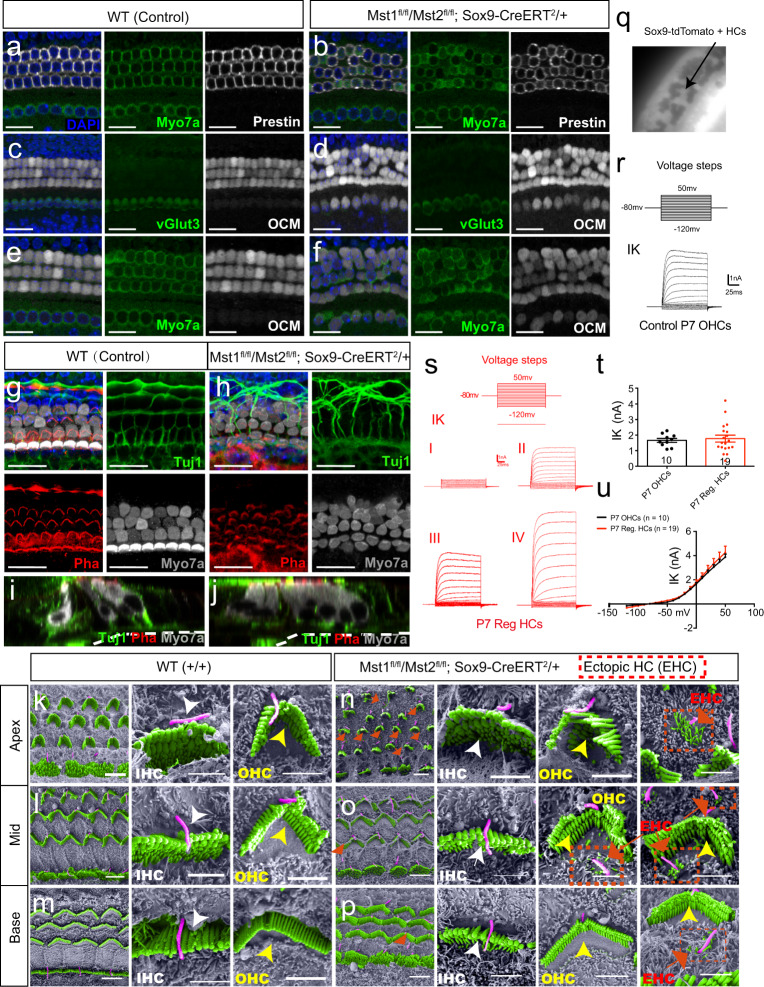
The supernumerary HCs induced by YAP nuclear translocation are functional but immature in vivo. **a**–**f** The supernumerary ectopic HCs in Mst1^fl/fl^/Mst2^fl/fl^; Sox9-CreERT^2^/+ mice were stained with the IHC marker vGlut3 and the OHC marker Prestin. Compared with WT mice, the newly generated HCs were labeled with OCM despite being located in the IHC and OHC region. **g** Phalloidin was used to label the stereocilia structure, and Tuj1 was used to label the nerve fiber. A “V”-shaped structure in OHCs and a “–”-shaped structure in IHCs labeled by phalloidin were observed and surrounded by orderly nerve fiber connections that were labeled by Tuj1 in P7 control mice. **h** In P7 Mst1^fl/fl^/Mst2^fl/fl^; Sox9-CreERT^2^/+ mice, almost all HCs—including regenerated HCs—had similarly shaped “V” and “–” structures labeled by phalloidin in the OHCs and IHCs, respectively. Tuj1 labeling showed 1–2 rows of inner HCs and 3–5 rows of outer HCs surrounded by irregular nerve fibers. **i**, **j** The sectional views of phalloidin and Tuj1 staining in control and transgenic mice at P7. **k**–**m** The representative view of ciliary structures from the apical to basal turn of the organ of Corti in WT mice under an electron microscope. **n**–**p** In littermate P7 Mst1^fl/fl^/Mst2^fl/fl^; Sox9-CreERT^2^/+ mice, immature hair bundles were detected with a decrease from the apical to basal turn. The enlarged views of IHC and OHC bundles in situ in control and Mst1/2 knockout mice and the immature ectopic hair bundles with slightly longer microvilli and a kinocilium from the apical to basal turn are shown on the right. **q** Mst1^fl/fl^/Mst2^fl/fl^; Sox9-CreERT^2^/+; Rosa26-tdTomato/+ mice were used to conduct the patch-clamp experiment. After tamoxifen injection at P1-2, cochleae were harvested at P7, thus the Sox9-tdTomato+ HCs could be observed under a microscope. **r** Representative outward K+ currents of WT P7 OHCs evoked by the voltage steps shown above*.* **s** Representative outward K+ currents of regenerated HCs in transgenic mice evoked by the same voltage steps shown above*.* **t** Mean ± SEM IK (Delayed rectifier potassium current) in response to 0 mV depolarization of WT P7 OHCs (*n* = 10) and regenerated HCs (*n* = 19)*.* **u** Mean ± SEM peak current–voltage relations of WT P7 OHCs and regenerated HCs. Data are shown as the mean ± SEM. Scale bars = 20 μm in (**a**–**f**, **g**, **h**). Scale bars = 5 μm in the low magnification and 2 μm in the high magnification, respectively in (**k**–**p**).

To identify the subtypes of the newly generated HCs in the *Mst1*^*fl/fl*^*/Mst2*^*fl/fl*^; Sox9-CreERT^2^/+ mice, vGlut3, Prestin, and Oncomodulin (OCM) were used to label the new supernumerary HCs. The Myosin7a+ cells in the OHC region expressed Prestin, while those in the IHC region expressed vGlut3 (Fig. 4a–f). A previous study showed that OCM labeling was mostly confined to OHCs, with some immunoreactivity labeling in IHCs in the early stages of development^33^. Strikingly, our results indicated that almost all new HCs were OCM+ in the transgenic mice, while OCM immunoreactivity was seen almost exclusively in the OHCs in the control group with very low expression among the IHC rows at P7. This result indicated that the newly generated HCs were different from the original HCs to some extent, and perhaps they were more immature.

Tuj1 was used to label the auditory neuronal projections surrounded by regenerated HC-like cells in vivo. One row of IHCs and three rows of OHCs were surrounded by nerve fiber connections in the control mice. In contrast, there were 1 or 2 rows of IHCs and 3–5 rows of OHCs in the transgenic mice, and the neurites around each HC grew out to the SR as seen in both coronal and axial sections (Fig. 4g–j). The stereocilia of the cochlear HCs were arranged as a recognizable “V”-shaped structure in OHCs and a “–”-shaped structure in IHCs in both groups, while the cilia of the regenerated HCs were slightly disorganized and out of alignment in the transgenic mice. Scanning electron microscopy was used to further visualize the cilia of newly regenerated HCs, and we found immature hair bundles in the new HCs at P7 (Fig. 4k–p). These results provide evidence for the status of newly generated HCs in vivo, which have similar but immature ciliary structures compared with WT mice. Additionally, the green fluorescence of the FM1-43FX dye was co-labeled with tdTomato after knockout of MST1/2 in Sox9+ SCs, which indicated that the new HCs had functional transduction channels (Fig. S8i).

A whole-cell patch-clamp experiment was performed in these supernumerary HCs. Sustained outward K+ currents were recorded in the newly generated HCs, similar to HCs over the course of development in the organ of Corti. These results indicated that regenerated HCs in vivo have some transduction channels and electrophysiological functionality (Fig. 4q–u).

### HC regeneration was enhanced by Hippo-off after HC damage in vitro

**Fig. 5:**
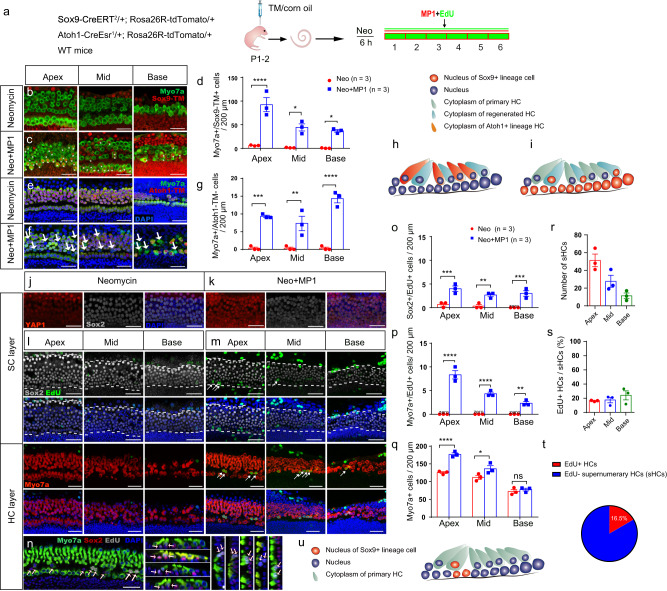
YAP nuclear translocation enhances HC regeneration after neomycin damage in vitro. **a** Sox9-CreERT^2^/+; Rosa26R-tdTomato/+ and Atoh1-CreEsr^1^/+; Rosa26R-tdTomato/+ mouse models were used to trace the SC and HC lineages. Tamoxifen was injected at P1 to activate tdTomato expression overnight, and the cochleae were harvested at P2 and then cultured in vitro. After neomycin exposure for 6 h, the cochlear explants were cultured with exogenous XMU-MP-1 or 0.5% DMSO for 5 days. **b**–**d** Compared with controls, the numbers of Myosin7a+/Sox9-tdTomato+ HCs were largely increased from the apical to basal turn in the XMU-MP-1-treated group analyzed with two-way ANOVA followed by Sidak’s multiple comparison test. **e**–**g** There were large numbers of Myosin7a+/Atoh1-tdTomato– HCs in the XMU-MP-1-treated groups compared to controls analyzed with two-way ANOVA followed by Sidak’s multiple comparison test. **h** The schematic diagram of Myosin7a+/Sox9-tdTomato+ HCs after XMU-MP-1 treatment. **i** The schematic diagram of Myosin7a+/Atoh1-tdTomato– HCs after XMU-MP-1 treatment. **j**, **k** The cochlear explants from P2 WT mice were harvested and then cultured. After neomycin exposure for 6 h, the explants were cultured with exogenous XMU-MP-1 or 0.5% DMSO for 24 h. Immunohistochemical staining results showed that the addition of XMU-MP-1 significantly increased the nuclear accumulation of YAP1 in Sox2+ SCs after neomycin injury. **l**, **m** After neomycin injury, the cochleae were cultured with DMSO or XMU-MP-1 for another 5 days, and 10 μM EdU was added the whole time to trace proliferating cells. In the control group with DMSO treatment, no proliferating HCs and very few SCs were detected. In the XMU-MP-1-treated group, there were some EdU+ SCs and HCs from the apical to basal turn. **n** The EdU+ HCs were mostly distributed in the tunnel between the inner and outer HCs, and they mostly appeared in pairs and stained with Sox2. **o**–**q** Compared with the control groups, there was a significant increase in the number of proliferating SCs and HCs after XMU-MP-1 addition in vitro analyzed with two-way ANOVA followed by Sidak’s multiple comparison test. The total number of HCs was significantly increased. **r**–**t** Only about 16% of HCs were EdU+ when compared with the increased proportion of HCs in the apical turn; sHC: supernumerary HC. **u** The schematic diagram of the mitotically regenerated HCs and proliferating SCs. *p < 0.05, **p < 0.01, ***p < 0.001, ****p < 0.0001. Data are shown as mean ± SEM. Scale bars = 20 μm.

The effect of XMU-MP-1 in neomycin-damaged cochleae in vitro is illustrated in Fig. 5a. We found that many more Myosin7a+/Sox9-tdTomato+ cells were generated with XMU-MP-1 treatment compared to the control cochleae (Fig. 5b, c). Numerically, more Sox9-tdTomato+/Myosin7a+ cells were found in the XMU-MP-1-treated group, with a decreasing pattern from the apex to the base (Fig. 5d), thus indicating that large numbers of Sox9+ SCs had directly differentiated into HCs in response to XMU-MP-1.

Atoh1, which is a member of the bHLH transcription family, is necessary and sufficient for early inner ear HC development^34^. In Atoh1-CreEsr^1^/+; Rosa26R-tdTomato/+ mice, we found that in the control group most of the HCs were Atoh1-tdTomato+, while in the transgenic mice the Myosin7a+/Atoh1-tdTomato– cells were in the tunnel between the IHCs and the OHCs (Fig. 5e–g). These results indicated that XMU-MP-1 significantly increased HC differentiation after neomycin insult, and the regenerated HCs were mostly differentiated from Sox9+ progenitors rather than from Atoh1-tdTomato+ HCs (Fig. 5h, i). The distribution of Sox9-tdTomato+ cells and Atoh1-tdTomato+ cells in the HC layer without neomycin damage were shown in Fig. S9a, b.

In WT mice, the addition of XMU-MP-1 significantly increased the nuclear accumulation of YAP1 in Sox2+ SCs after neomycin injury (Fig. 5j, k) and no Myosin7a+/EdU+ cells but a few Sox2+/EdU+ cells were found in the neomycin control group. Conversely, significantly increased numbers of both Sox2+/EdU+ and Myosin7a+/EdU+ cells were found in the XMU-MP-1-treated cochleae (Fig. 5l–q). Interestingly, almost all of the Myosin7a+/Sox2+/EdU+ cells were present together in pairs. In addition, we found that most of the mitotically proliferating HCs were located between the IHCs and OHCs, which is the region where the precursor cells are enriched^7^ (Fig. 5n). These results suggested that regulation of Hippo signaling might be a context-dependent SC proliferative regulator that induces significantly more SC proliferation in the case of damaged cochleae. Furthermore, mitotically generated HCs (EdU+/Myosin7a+/Sox2+) were clearly increased compared to the intact cochleae (Fig. S9c, d), but these were still not the major cell type resulting from YAP nuclear translocation-induced HC regeneration in vitro (Fig. 5q–u).

### Overexpression of *Yap* initiated supernumerary HCs

As the above results indicated, YAP protein was increased in the SC nucleus after neomycin damage (Fig. 1g–i). Additionally, XMU-MP-1 promoted YAP nuclear translocation in SCs in a dose-dependent manner not only in the intact condition (Fig. 2b–g), but also in the neomycin damage condition (Fig. 5j, k), which provided further strong evidence that YAP nuclear translocation is associated with HC regeneration.

**Fig. 6:**
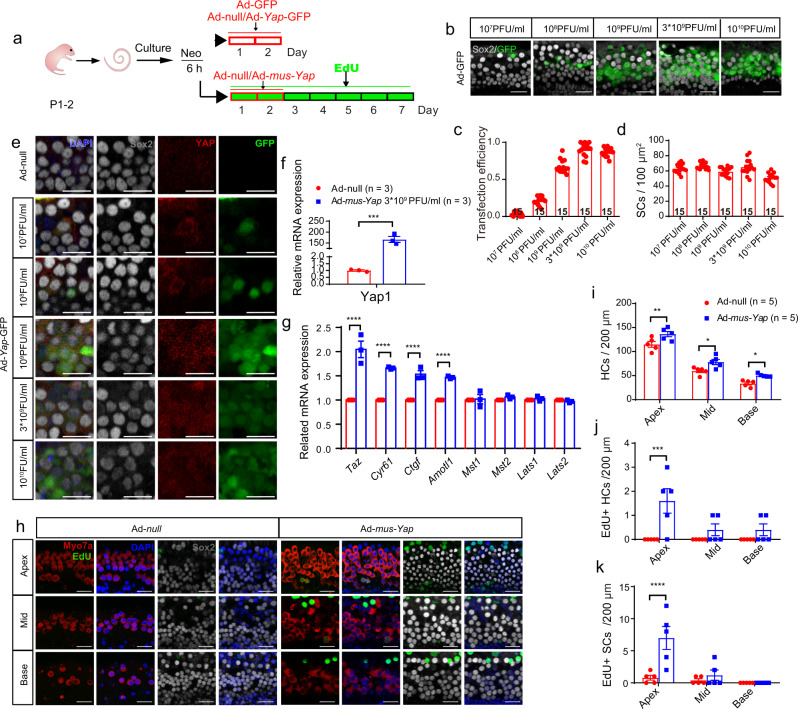
Overexpression of YAP initiated supernumerary HCs. **a** After neomycin exposure for 6 h, the cochlear explants were cultured with exogenous Ad-GFP/Ad-Yap-GFP for 48 h, and the transfection efficiency based on the GFP fluorescence was analyzed in Sox2+ SCs in the SR. **b**, **c** GFP+/Sox2+ cells were counted, and the results showed that the transfection efficiency increased with the increasing adenovirus concentrations (10^7^, 10^8^, 10^9^, 3 × 10^9^, and 10^10^ PFU/ml). **d** The numbers of Sox2+ SCs treated with different concentrations of adenovirus were calculated. It was found that the number of SCs decreased slightly at high concentration. **e**, **f** To upregulate the expression of *Yap*, the cochlear explants after neomycin insult for 6 h were treated with Ad-Yap-GFP for 48 h. One-sample *t* test was performed and showed that YAP was significantly increased with a concentration of 3 × 10^9^ PFU/ml adenovirus, and the distribution of YAP in the nucleus was significantly increased. **g** The relative mRNA expression level of Hippo signaling and downstream target genes after Ad-Yap-GFP treatment, and two-way ANOVA followed by Sidak’s multiple comparison test was performed to show the significant differences. **h**–**k** After neomycin exposure for 6 h, the cochlear explants were cultured with Ad-mus-Yap1 or Ad-null for 7 days, and 10 μM EdU was added the whole time to trace proliferating cells. In the control group with Ad-null treatment, no proliferating HCs or SCs were detected. In the Ad-mus-Yap1 treatment group, there were significantly more EdU+ SCs and HCs from the apical to basal turn analyzed with two-way ANOVA followed by Sidak’s multiple comparison test. Compared with controls, the number of HCs was significantly increased from the apical to the basal turn analyzed with two-way ANOVA followed by Sidak’s multiple comparison test. *p < 0.05, **p < 0.01, ***p < 0.001, ****p < 0.0001. Data are shown as the mean ± SEM. Scale bars = 20 μm in (**b**) and (**h**), Scale bars = 10 μm in (**e**).

We next tried to determine whether turning off Hippo initiated the generation of supernumerary HCs through stimulation of YAP nuclear translocation. Adenovirus was used as a vector for efficient transfection of the cochlear explants, and recombinant adenovirus (Ad-YAP, and Ad-YAP-GFP) were generated to overexpress Yap1. The optimal concentration of 3 × 10^9^ PFU/ml adenovirus was selected for further analysis (Fig. 6a–g). Significantly increased numbers of both Myosin7a+ cells and Myosin7a+/EdU+ cells were found in the Ad-YAP-treated cochleae (Fig. 6h–k). These results suggested that direct differentiation dominated HC regeneration compared to mitotically generated HCs.

### Notch pathway inhibition and Hippo-off synergistically promoted HC regeneration

**Fig. 7:**
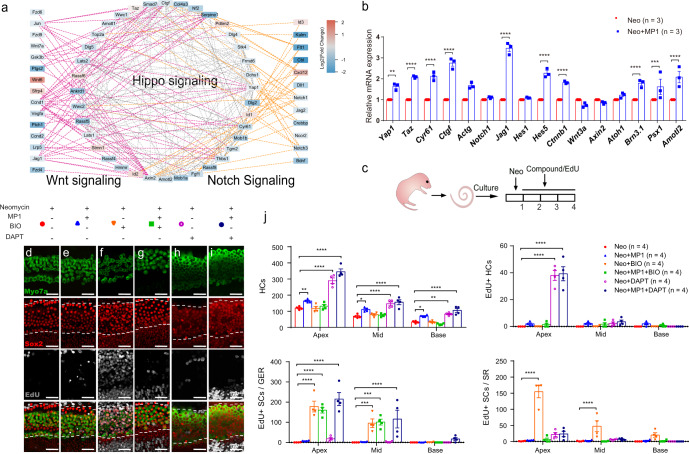
Notch pathway inhibition works synergistically with Hippo-off to promote HC regeneration. **a** Protein–protein interaction network analysis of differentially expressed genes of the Hippo, Notch, and Wnt signaling pathways. Purple lines indicate interactions between Hippo and Wnt genes, and orange lines indicate interactions between Hippo and Notch genes. The color of the nodes indicates the gene expression fold change. **b** The cochlear explants from P2 wild-type mice were harvested and then cultured. After neomycin exposure for 6 h, the explants were cultured with XMU-MP-1 or DMSO for 24 h. Related mRNA expression levels between XMU-MP-1 treated groups and controls are shown, and two-way ANOVA followed by Sidak’s multiple comparison test was performed to show the significant differences. **c** The cochlear explants from P2 WT mice were harvested and then cultured. After neomycin exposure for 6 h, the explants were cultured with exogenous compounds (including XMU-MP-1, BIO, or DAPT) or 0.5% DMSO for 3 days, and 10 μM EdU was added the whole time to trace proliferating cells. **d**–**g** The total numbers of HCs and EdU+ HCs were not significantly different between the XMU-MP-1-treated and XMU-MP-1/BIO-treated groups. **e**, **h**, **i** The number of regenerated HCs was significantly increased in the XMU-MP-1/DAPT-treated group when compared to the XMU-MP-1-treated group. j Two-way ANOVA followed by Dunnett’s multiple comparisons test was performed and showed that the upregulation of Wnt signaling by BIO could promote the proliferation of SCs. However, XMU-MP-1/DAPT co-regulation generated more HCs, including EdU+ HCs, and large numbers of proliferating SCs were found in the GER. *p < 0.05, **p < 0.01, ***p < 0.001, ****p < 0.0001. Data are shown as the mean ± SEM. Scale bars = 20 μm.

In our previous study, we found that coordinated regulation of Wnt activation and Notch inhibition could promote HC regeneration in the postnatal mouse cochlea^7,35^. By analyzing the transcripts of cochleae after neomycin injury, protein interaction network analysis using STRING indicated that the expression of Hippo, Notch, and Wnt signaling pathway-related genes varied remarkedly in the regeneration process (Fig. 7a). The RT-qPCR results indicated that Hippo inhibition was accompanied by upregulation of genes related to HC transformation (*Atoh1*, *Brn3.1*), the Notch signaling pathway (*Jag1*, *Hes1* and *Hes5*), and *Yap/Taz* and its downstream effector genes (*ACTG* and *Amotl2*) in the XMU-MP-1-treated cochleae after neomycin insult (Fig. 7b).

Furthermore, we investigated the synergistic/antagonistic effects of turning off Hippo with other signals (Fig. 7c). Some proliferating SCs and regenerated HCs in the SR were observed when Hippo signaling was turned off alone (Fig. 7d, e, j), while large numbers of proliferating SCs (in the SR and GER) and nearly no direct-differentiated HCs were observed when the Wnt pathway was activated alone (with BIO) (Fig. 7f, j). We observed some proliferating SCs and regenerated HCs in the SR when turning off Hippo was combined with Wnt activation (Fig. 7g, j). The proliferation of SCs in the combined Hippo inactivation/Wnt activation group was significantly inhibited compared with Wnt pathway activation alone but resembled that in Hippo signaling pathway regulation alone, especially in the SR. Meanwhile, the directly differentiated HCs were not affected by Wnt activation, and their numbers were similar to those seen upon turning off Hippo signaling alone. These results indicate that there is no obvious synergistic effect of Wnt activation and Hippo inactivation.

Finally, some proliferating SCs and large numbers of directly differentiated HCs were observed in the SR when the Notch pathway was inhibited alone (Fig. 7h, j), and many proliferating SCs and regenerated HCs were observed when upregulation of Hippo was combined with Notch suppression (with DAPT) (Fig. 7I, j). Specifically, the proliferation of SCs was significantly reduced in the SR compared to that seen with Notch inhibition alone, but SC proliferation was mainly found in the GER. Also, the number of directly differentiated HCs increased significantly compared with turning off Hippo alone. These results suggest that there was a significant synergistic effect of Notch signaling on the Hippo pathway, especially in promoting the direct differentiation of SCs into HCs. In conclusion, Hippo/Wnt/Notch regulation, especially the combination of Hippo and Notch, appears to play an important role in inner ear HC regeneration.

## Discussion

Hippo-YAP signaling is an important regulator for tissue homeostasis, organ size, and stem cells^36^. Our previous RNA-sequencing analysis between neomycin-treated and intact Lgr5+ progenitors revealed that *Bmpr2*, *Wnt7a*, and *Fzd7* were upregulated, while *Id1*, *Id2*, and *Id3* were downregulated among the Hippo pathway genes^37^. In the present study, we focused on Hippo signaling, and 56 Hippo-related genes were shown to be associated with HC damage and self-repair processes. Our results indicated that the biological processes of damage and repair existed simultaneously in the damaged cochleae.

As a context-dependent regulator, Hippo signaling is involved in cell proliferation, differentiation, and apoptosis in various cell subtypes^38^. YAP, as the effector molecule of the Hippo pathway, is required for certain cell specifications during the very early phases of embryonic development^20,39^. Gnedeva et al. characterized the role of YAP in maintaining the proliferative state of organ of Corti progenitors before the establishment of the postmitotic prosensory domain^25^. In response to damage, Hippo-independent signaling may promote the effect of YAP to prevent malignant transformation^40^. Here we showed the intracellular pattern of YAP in neonatal mice (P2), with YAP mostly distributed in the cytoplasm and only weak signals in the cell nucleus. The YAP cytoplasmic-nuclear translocation was initiated by neomycin. These results suggest that YAP nuclear accumulation might play an important role in the process of HC damage and self-repair and that regulating YAP nuclear accumulation might be crucial for HC regeneration after damage.

Hippo signaling plays an important role in maintaining homeostasis and maintaining organ size through biochemical feedback loops^19,41^. It has been widely reported that Hippo signaling regulates organ size and reaches homeostatic levels by modulating proliferation, survival, and differentiation in the liver tissue of mammals^42–45^. A recent study highlighted the role of the *Yap/Tead* transcription factor complex, which is the effector molecule of the Hippo pathway, in maintaining inner ear progenitor cells during development^25,26^. Other studies have indicated that activation of YAP in postnatal cardiomyocytes can stimulate cardiomyocyte expansion in therapeutic myocardial regeneration^46,47^. Here we reported the role of *Yap* expression and localization after HC damage in neonatal mammalian cochlear cells. The downstream genes of the Hippo pathway were upregulated by XMU-MP-1, and this might be closely related to the SC reprogramming and HC regeneration process.

It has been reported that loss of upstream kinases or activation of YAP can regulate the growth and cell fate decisions in progenitor cells^39,48^. To investigate the effect of the Hippo signaling pathway in the inner ear, we pharmacologically inhibited the Hippo signaling pathway thus inducing YAP nuclear translocation in vitro, and we further employed *Mst1/2* conditional knockout mice to verify the role of Hippo inhibition in the inner ear in vivo. We observed a number of proliferating SCs, which was consistent with their known functions in regulating the growth and development of other organs^20^. More importantly, proliferative generation and direct differentiation of HCs from SCs was observed in transgenic mice in vivo, and YAP nuclear translocation could replenish the neomycin-damaged HCs in vitro. Specifically, over 80% of the increased HCs were non-proliferative, illustrating that direct differentiation was the dominant process of HC generation in response to Hippo inhibition and that this might be closely correlated with the limited proliferation of SCs. In addition, we used the Mst1^fl/fl^/Mst2^fl/fl^; Sox9-CreERT^2^/+; Rose26-tdTomato/+ mouse lineage to show that almost all the supernumerary HCs originated from Sox9+ SCs. These results showed that Hippo inhibition could promote SC proliferation and induce supernumerary HC generation by maintaining the dynamic balance among different cell subtypes in the inner ear. Furthermore, our results demonstrated that YAP nuclear translocation could initiate more mitotic generation and direct differentiation into HCs to replenish the neomycin-damaged HCs, which might be a feedback mechanism regulated by Hippo signaling. HC regeneration resulted in reduced numbers of SCs and thus induced SC proliferation, which in turn led to new HC generation^49^. These processes might not exist independently but might instead influence each other through a dynamic balance within a closed loop.

It is notable that neomycin damage together with inactivation of MST1/2 kinases by XMU-MP-1 dose-dependently induces sensory cell fate acquisition mostly through direct differentiation of SCs, accompanied by some EdU+ HCs. However, in the undamaged cochleae, with MST1/2 knockout by transgenic method, we failed to find extensive cell proliferation except for a small number of proliferated SCs or immature supernumerary HCs. We hypothesized that this contradiction might be due to the difference between the neomycin-damaged and intact micro-environment. As reported previously, damage might trigger some HC regeneration mechanisms, especially in non-mammalian species such as birds and fish^50,51^. In contrast, mammalian HCs cannot be spontaneously regenerated. In the present study, we conclude that Hippo-off through MST1/2 inhibition or *Yap* overexpression induces YAP nuclear accumulation, especially in SCs, which induces a small number of supernumerary HCs without neomycin, or the numbers of HCs are increased with neomycin injury in vitro. Our results indicate that in the damaged condition, Hippo signaling might remove some barriers in SCs to drive them to directly differentiate into HCs. This mechanism needs to be further investigated in the future.

Structurally, there are neural connections between HCs and the auditory centers of the brain. In the Mst1^fl/fl^/Mst2^fl/fl^; Sox9-CreERT^2^/+ mouse model, we found that newly generated HCs were able to promote neurite outgrowth. Importantly, the formation of nerve fibers was observed in vivo, which supports the hypothesis that the generation of supernumerary HCs in situ attracts connections between HCs and neurons, and this suggests that the new HCs could reconstruct neural connections and thus have the potential for restoring hearing loss. However, the regenerative ability of HCs is decreased rapidly with age in vivo and in vitro^12,13^. Future work should therefore focus on understanding the underlying mechanisms behind these processes in order to develop new strategies for recovering hearing in mammals.

In a previous study, mechano-activation of YAP/TAZ was reported to promote epidermal stemness through inhibition of Notch signaling^52^, and a large body of work has shown that the Hippo, Wnt, and Notch signaling pathways do not work independently and may influence each other^47,53–55^. It was also reported that activation of Wnt signaling promoted SC proliferation and that inhibition of Notch signaling stimulated HC regeneration^7,35^. Here, we investigated the influence of these two classical signaling pathways on the functions of Hippo in promoting regeneration. The cross-talk between YAP and Notch signaling influences cell development, cell differentiation, cell fate decisions, morphogenesis, inflammation, epithelial stromal interactions, and large-scale gene oscillations^56^. It has been reported that YAP/TAZ activates Jagged-1 expression and thus promotes Notch-dependent smooth muscle differentiation in the neural crest^55^. In our study, there was no obviously increased SC proliferation or HC regeneration in the SR upon combined regulation of the Wnt and Hippo pathways compared with the regulation of Hippo alone. However, there was greater SC proliferation and more HC regeneration in the SR in response to the combined regulation of the Notch and Hippo pathways compared to regulating a single signaling pathway at a time. These results suggest that inhibiting the Notch pathway has a synergistic and positive regulatory effect on Hippo signaling, and we will pursue further research on the sequential regulation of these pathways in order to directly verify these assumptions.

In conclusion, YAP nuclear accumulation in response to Hippo inhibition can promote mild SC proliferation and can induce supernumerary HC generation in neonatal mice, and it especially promotes direct SC trans-differentiation together with neomycin damage mainly in the apical to middle turns of the cochlea. We provide ample evidence for the positive effect of Hippo inhibition on HC regeneration and show the synergistic effect with the Notch and Wnt signaling pathways, which will hopefully lead to new strategies for promoting SC proliferation, HC regeneration, and reconnection with neurons in the mammalian inner ear.

## Methods

### Animals

All transgenic mice (Sup Table 1) were obtained from the Jackson Laboratory. For Cre activation, 15 µl tamoxifen (Sigma) dissolved in corn oil was intraperitoneally injected into P1–2 mice (40 mg/ml). For detecting proliferating cells, 20 µl of the thymidine analog EdU (Click-iT EdU Imaging Kit; Invitrogen) was intraperitoneally injected into P2–7 neonatal mice (5 mg/ml).

We complied with all relevant ethical regulations for animal testing and research. All animal experiments were performed according to protocols approved by the Institutional Animal Care and Use Committee of Fudan University, and all efforts were made to minimize the number of animals used and to prevent their suffering.

### Genotyping and RT-qPCR

Transgenic mice were genotyped with genomic DNA extracted from tail tips using the Viagen DirectPCR DNA Extraction System (Viagen). The genotyping primers are provided in Supplementary Table 2.

Total RNA was extracted from the cochlea with the anlage of the stria vascularis, the modiolus, and the tectorial membrane removed using TRIzol (Ambion). cDNA synthesis and qPCRs were performed with the PrimeScript^TM^ II 1st Strand cDNA Synthesis Kit (Takara) and TB Green^TM^ Premix Ex Taq^TM^ II (Takara). Each PCR was carried out in triplicate, and the relative quantification of gene expression was performed using the ΔΔCT method with *Gapdh* as the endogenous reference. Primer pairs were designed using the online Primer3 software, and sequences are provided in Supplementary Table 3.

### Tissue culture

Cochleae of P2 neonatal mice were isolated under sterile conditions with the anlage of the stria vascularis, modiolus, and tectorial membrane removed with fine forceps, then placed onto 10 cm coverslips pre-coated with Corning® Cell-Tak™ Cell and Tissue Adhesive. Whole organs were cultured in DMEM/F12 medium supplemented with N2/B27 (Invitrogen) and ampicillin sodium solution (Sangon Biotech) in four-well Petri dishes. For HC insult, neomycin (1 mM, Sigma) was added after culturing for 12 h, and 10 µM EdU was added to detect proliferating cells. In the cochlear culture model in vitro, 1 mM and 5 mM XMU-MP-1 (Selleck), 5 µM DAPT (N-[N-(3,5-Difluorophenacetyl)-L-alanyl]-S-phenyl glycine t-butylester, a γ-secretase secretase inhibitor), and 5 µM BIO (6-Bromoindirubin-3’-oxime) were added to regulate the biological activity of the Wnt and Notch signaling pathways.

### Adenovirus incubation

To construct the recombinant Yap-overexpression adenovirus vectors, pAd/CMV/ V5-DEST Gateway® Vector, pAd/PL-DEST Gateway® Vector, and 293 cell lines (Invitrogen, Carlsbad, California, USA) were prepared. Mouse cDNA for *Yap* (NM_001171147CDS) was inserted into the adenoviral vectors. The digested vector and insert segments were ligated with T4 DNA ligase. Extensive extraction and packaging of the viral plasmids was performed, followed by collection and amplification. Finally, recombinant adenovirus with a titer of 1.2 × 10^10^ PFU/ml was obtained. For cochlear explant transfection, recombinant adenovirus (Ad-null, Ad-GFP, Ad-YAP, and Ad-YAP-GFP) was added to the medium and allowed to incubate for 48 h. The concentrations of recombinant virus were 10^7^, 10^8^, 10^9^, 3*10^9^, and 10^10^ PFU/ml. Tissues were prepared for immunohistochemical labeling and analyzed at 7 days after adenovirus incubation.

### Immunohistochemistry

Tissues were fixed with 4% paraformaldehyde in 0.1 M phosphate-buffered saline (PBS) for 10 min. After washing with PBS three times, the tissues were immersed in blocking solution consisting of 10% donkey serum and 1% Triton-X100 in PBS at pH 7.4 for 1 h at room temperature. The primary antibodies and secondary antibodies were diluted in 1% Triton-X100 in PBS overnight at 4 °C in a humidified chamber. After washing with PBS, tissues were mounted in antifade fluorescence mounting medium and cover-slipped. The primary antibodies used in our experiments were rabbit anti-Myosin7a (1:500 dilution) (Proteus Biosciences), goat anti-Sox2 (1:500 dilution) (Abcam), and mouse anti-YAP (1:50 dilution) (Santa Cruz). HC bundles were labeled with phalloidin (1:500 dilution) (Life Technologies), nerve fibers were labeled with Tuj1 (1:500 dilution) (Abcam), and nuclei were labeled with DAPI (1:500 dilution) (Sigma). The FM1-43 uptake experiment was performed using FM1-43FX, a fixable analog of the FM1-43 membrane stain (1:500 dilution) (Invitrogen).

### Scanning electron microscopy (SEM)

The cochlea was perfused immediately with 4% paraformaldehyde after the mouse was anaesthetized. The tissues were then immersed in 2.5% glutaraldehyde for SEM. The SEM samples were post-fixed in 1% phosphate-buffered OsO_4_, dehydrated in a graded ethanol series, dried, mounted onto an aluminum sheet, and sprayed with gold/palladium. SEM was performed using a ZEISS GeminiSEM 300 (Germany). The photos are shown in pseudo-color.

### Electrophysiology

To assess the functions of native HCs in WT mice and newly generated HCs in Mst1^fl/fl^/Mst2^fl/fl^; Sox9-CreERT^2^/+; Rosa26-tdTomato/+ mice, we performed patch-clamp recordings in excised organs of Corti. For these electrophysiological experiments, cochleae from P7 WT mice or transgenic mice were freshly dissected in DMEM/F12 and then bathed in extracellular solution containing (in mM): 135 NaCl, 5.8 KCl, 1.3CaCl_2_, 0.9 MgCl_2_, 0.7 NaH_2_PO_4_⋅H_2_O, 10 HEPES, 5.6 D-glucose, 2 Na-pyruvate (309 mOsm, pH was adjusted to 7.40 with NaOH). The apical and middle turns of the cochleae were then immobilized on the glass with cell-TeK (Sigma) and transferred to the recording chamber. Random native OHCs, SCs near the IHCs, and regenerated HCs in the apical turn were selected for recording.

Whole-cell patch-clamp recordings were made using Patchmaster software (HEKA Electronics) and the EPC10/2 amplifier (HEKA Electronics, Lambrecht Pfalz, Germany). Patch pipettes were 3–5 MΩ pulled from borosilicate glass capillaries (Sutter). The pipette filling solution contained (in mM): 112 K-methane sulfonate, 20 KCl, 10 HEPES, 2 ethylene glycol tetraacetic acid, 10 Na-phosphocreatine, 3 Mg-ATP, 0.5 Na-GTP (285 mOsm, pH was adjusted to 7.40 with KOH). The specimens were observed in an up-right microscope (Olympus) with Nomarski differential interference contrast optics (60× water-immersion objectives).

Cells were held at –80 mV for voltage clamp, and for total K+ current recording we used voltage steps from −120 mV to +50 mV at 10 mV per step. Experiments were carried out at room temperature, and voltages were corrected offline for a measured liquid junction potential of 6 mV (K internal).

Electrophysiology data were analyzed with Igor software (Pro 6.22) and GraphPad Prism6. For K+ current analysis, data were accepted if *R*_*s*_ was no greater than 20 MΩ. The data are reported as means ± SEM.

### RNA-sequencing and analysis

Six to eight cultured cochleae were collected to isolate the total RNA using the AllPrep DNA/RNA/Protein Mini Kit (QIAGEN) as reported in the previous study^32^.

Preliminary processing of raw reads was performed using Casava 1.8 (Illumina), and adapters and low-quality bases were trimmed using trimmomatic (version 0.23). Trimmed reads for each sample were aligned to the *Mus musculus* UCSC mm10 genome using Hisat2^50^. Aligned reads were counted with HTseq (doi:10.1093/bioinformatics/btu638) according to UCSC mm10 annotation. Differential expression analysis between conditions was performed using the DESeq2 R package (Love et al., 2014). Genes with an adjusted p-value (padj) < 0.05 and fold change >1.5 were considered to be differentially expressed. Gene Ontology analysis of significantly differentially expressed genes was done with DAVID (http://david.abcc. ncifcrf.gov/home.jsp). The Pheatmap R package was used to generate heatmaps. Protein–protein interaction (PPI) network analysis of differentially expressed genes in the Hippo, Notch, and Wnt signaling pathways was performed using STRING software.

In the functional enrichment analysis. The cutoff of 0.1 for the false discovery rate was used to include more potentially involved pathways.

### Western blot analysis

Total protein of the cochleae was isolated with RIPA (Beyotime) lysis buffer supplemented with PMSF (Beyotime). Protein concentrations were measured using a BCA protein assay kit (Beyotime), and proteins were separated on a BeyoGel™ Plus Precast PAGE Gel for Tris-Gly System (Beyotime) and transferred onto polyvinylidene difluoride membranes (Beyotime). The membranes were blocked with 10% nonfat dried milk in Tris-buffered saline with Tween 20 (TBST, 50 mM Tris-HCl (pH 7.4), 150 mM NaCl, and 0.1% Tween 20) for 1 h at room temperature and then blotted overnight with primary antibodies at 4 °C. The primary antibodies were as follows: rabbit anti-Yap (1:1000 dilution) (CST), rabbit anti-phospho-Yap (1:1000 dilution) (Ser127) (CST), rabbit anti-LATS1 (1:1000 dilution) (CST), rabbit anti-phospho-LATS1 (1:1000 dilution) (Thr1079) (CST), rabbit anti-Cyr61 (1:500 dilution) (Santa Cruz), and goat anti-CTGF (1:500 dilution) (Santa Cruz). All blots derive from the same experiment and that they were processed in parallel.

### Image acquisition, cell counting, and statistical analysis

An SP8 confocal microscope was used to acquire florescent images. All of the images were labeled and digitally processed using ImageJ, Adobe Photoshop CS6, and Illustrator CC. The schematic diagram was drawn using the CDR software. The cell counts in the fluorescent images were performed using ImageJ. Total numbers of HCs, SCs, EdU+ cells, and double-stained cells were counted in 200 µm regions of the cochlea from the apical, middle, and basal turns.

Statistical analyses were performed using GraphPad Prism software. One-sample *t* tests and two-tailed unpaired Student’s *t* tests were used to determine statistical significance when comparing two groups, and one-way and two-way ANOVA were used when comparing more than two groups. A value of *p* < 0.05 was statistically significant. Data are shown as the mean ± SEM.

### Reporting summary

Further information on research design is available in the Nature Research Reporting Summary linked to this article.

## Supplementary information

REPORTING SUMMARY

Supplementary Information

## Data Availability

The data that support the findings of this study are available from the corresponding author upon reasonable request. The RNA-sequencing analysis data have been deposited in the GEO public database repository (accession number GSE213784).
