# Supplementary material for: Loss of Mst1/2 activity promotes non-mitotic hair cell generation in the neonatal organ of Corti
Source: NPJ Regen Med. 2022 Oct 25;7:64. doi: 10.1038/s41536-022-00261-4 (PMC9592590; doi:10.1038/s41536-022-00261-4)
Supplement: Supplementary file 2 — Supplementary Information [file 41536_2022_261_MOESM2_ESM.pdf]

Supplementary figure legends:

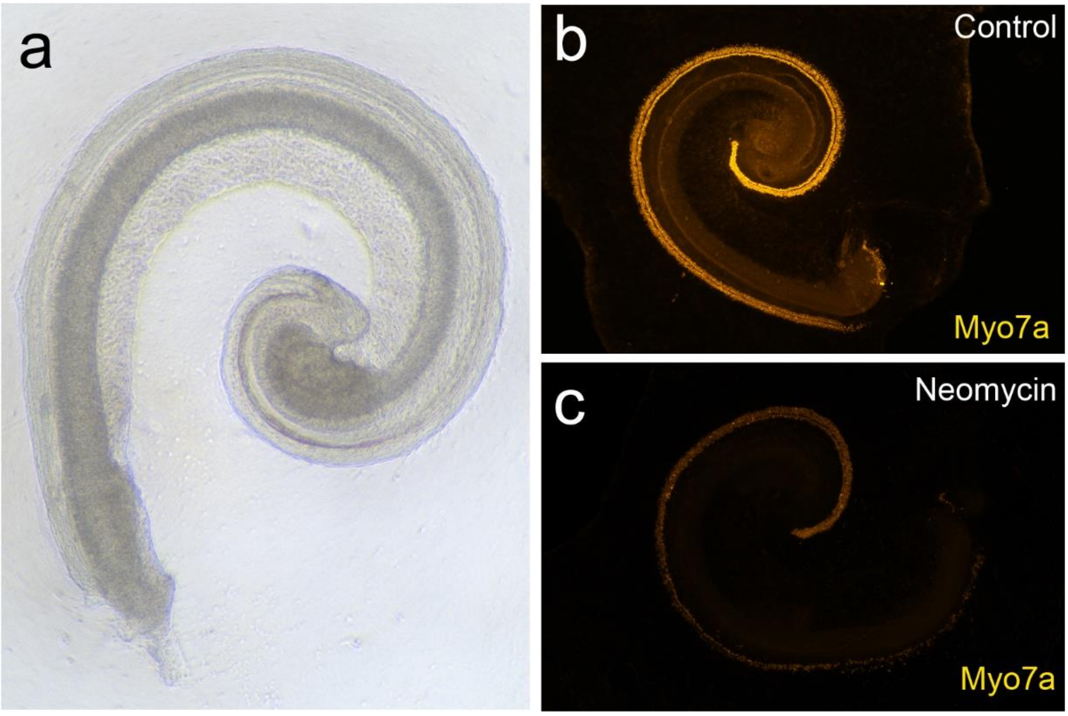

Supplementary figure 1: **Panoramic view of the cochlear explants cultured in vitro.** *a* Morphology of a cultured cochlea from a P2 neonatal mouse under a light microscope. *b-c* The distribution of Myosin7a+ HCs of the entire uninjured (*b*) and neomycin-treated (*c*) cochlea under a light microscope.

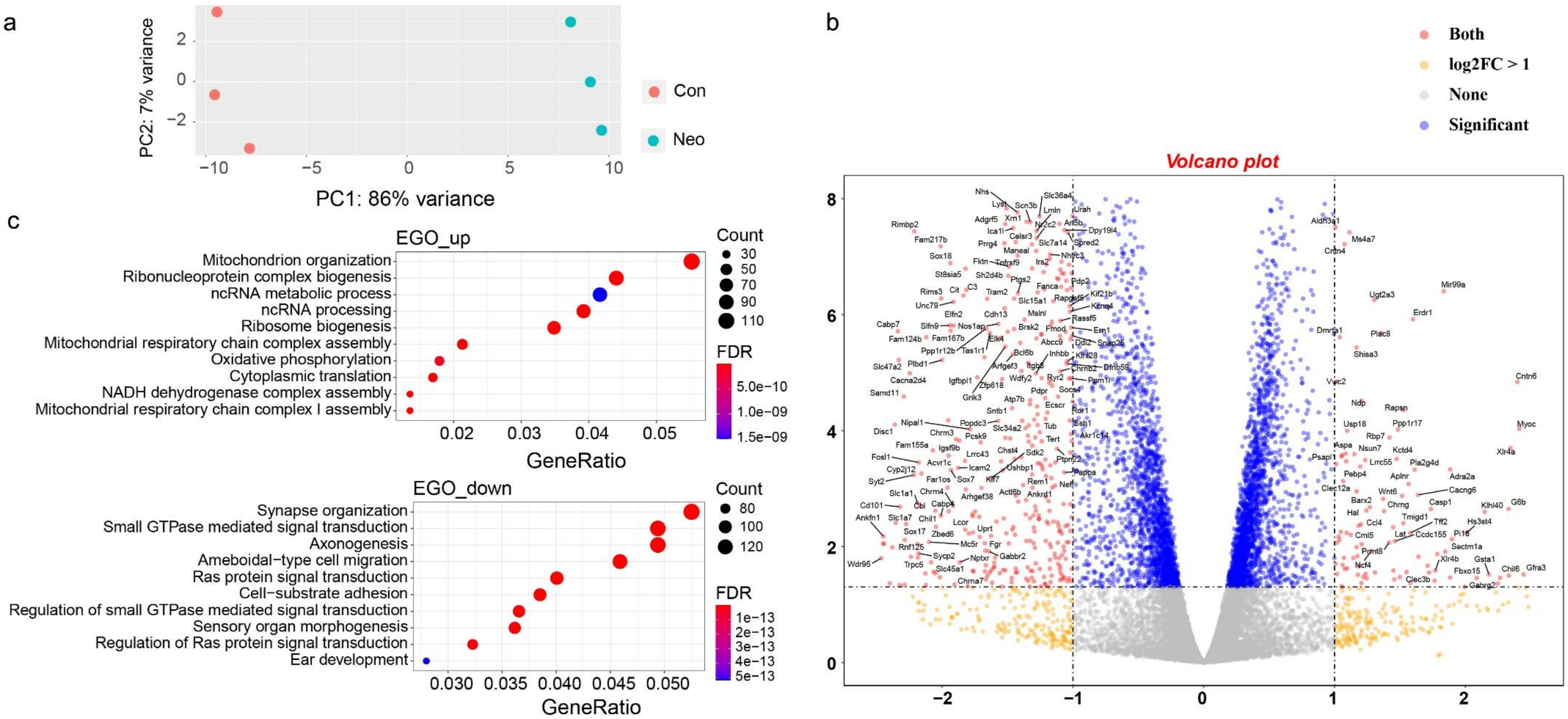

Supplementary figure 2: Global comparison of gene expression between control and damaged neonatal mammalian cochleae *in vitro*. *a* Principal component analysis of RNA-seq data from control and damaged cochleae demonstrated that the three replicates clustered tightly with each other (PC1 = 64%, PC2 = 21%). *b* The volcano plot showing the intersection and union of the gene expression between the two groups. *c* The top 10 GO terms for up and down-regulated genes between the two groups.

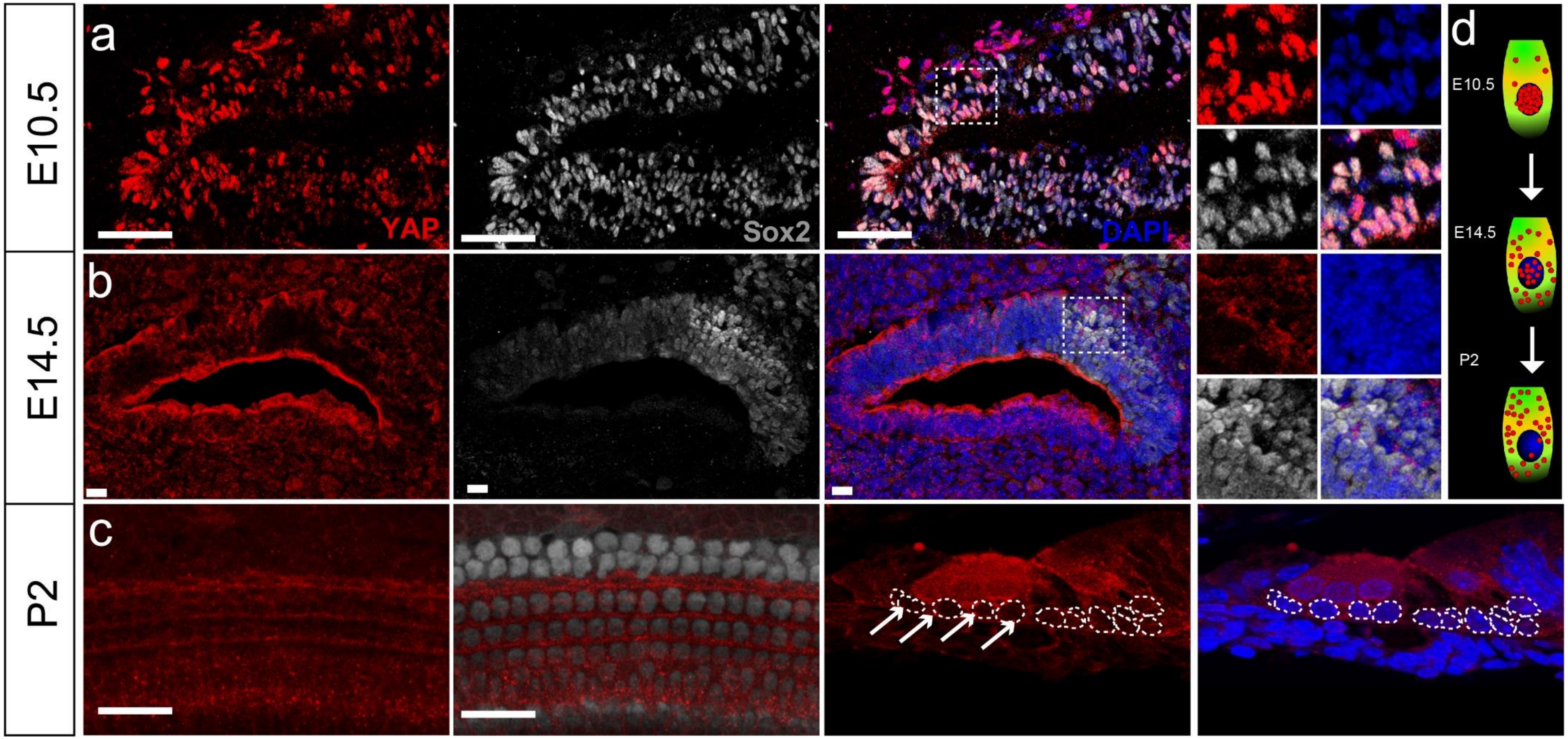

Supplementary figure 3: The expression pattern of YAP with the development of the cochlea. *a* YAP immunohistochemical staining at E10.5 showed significant nuclear accumulation. *b* The level of YAP nuclear distribution significantly decreased at E14.5. *c* YAP was mostly expressed in the cytoplasm of SCs at P2. *d* The schematic diagram of YAP nuclear accumulation from embryos to newborns. Scale bars = 20 μm.

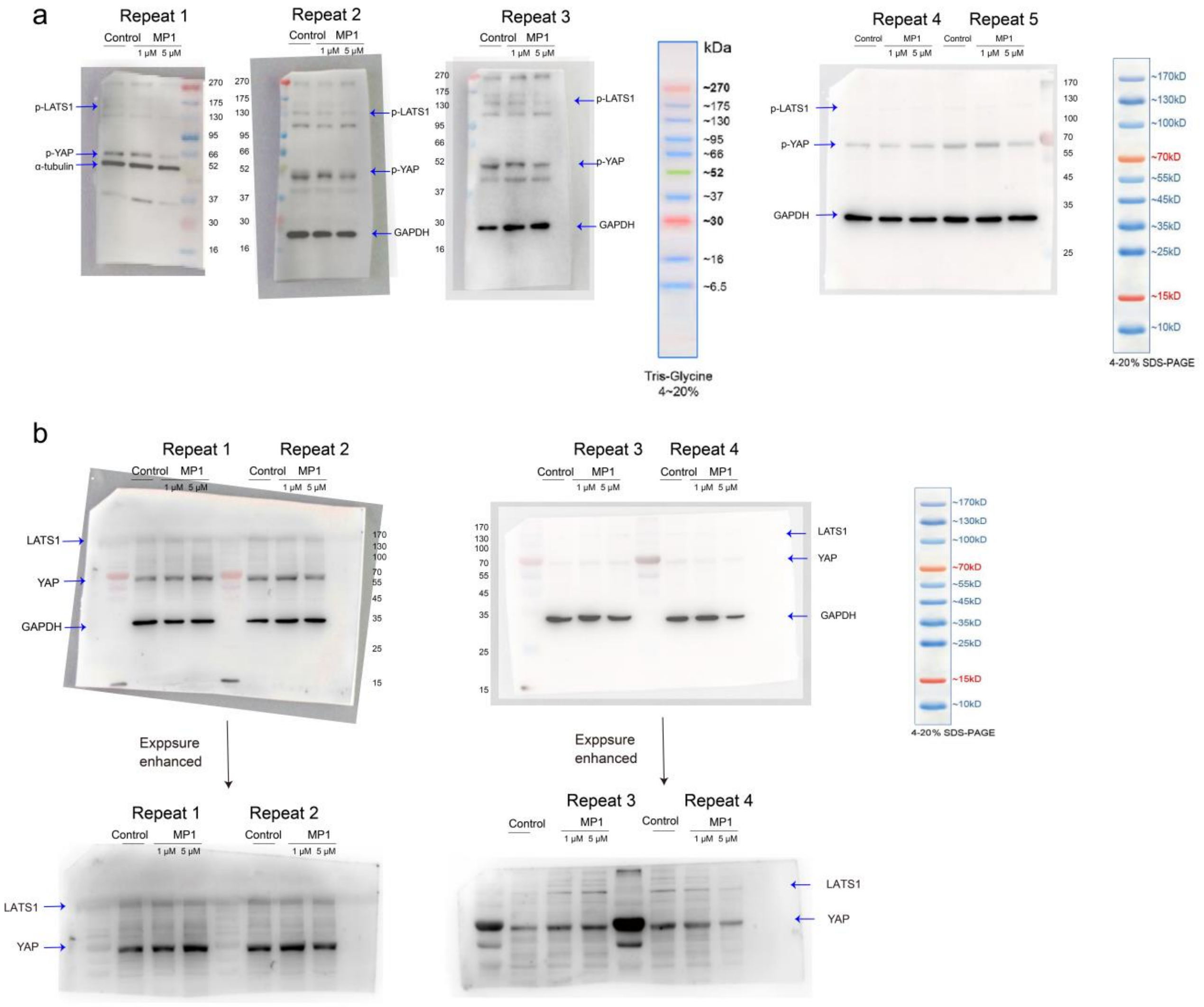

Supplementary figure 4: Unprocessed blots in Figure 2j used for statistical analysis. *a* Western blotting analysis was performed on extracts of control, 1 μM, and 5 μM XMU-MP-1–treated cochleae using p-LATS1, p-YAP, and GAPDH. Chromatograms of the molecular weight standard for color pre-dyed proteins (6.5-270 kDa). *b* Western blotting analysis was performed on extracts of control, 1 μM, and 5 μM XMU-MP-1–treated cochleae using LATS1, YAP, and GAPDH. Chromatograms of the molecular weight standard for color pre-dyed proteins (10-170 kDa).

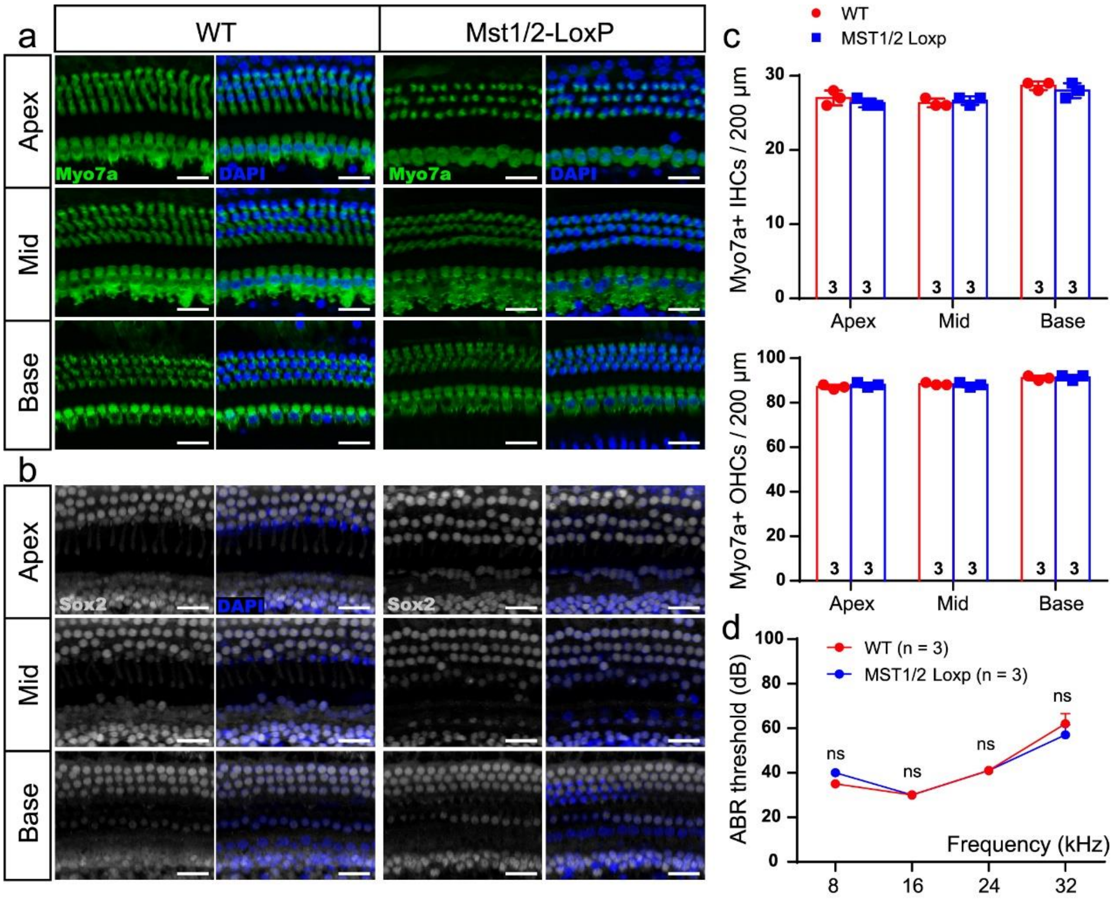

Supplementary figure 5: Introducing Mst1/2 loxP sites does not affect the phenotype of the cochlea or influence hearing. *a-b* Cochleae of P25 WT and $Mst1^{fl/fl}/Mst2^{fl/fl}$ adult mice were dissected and immunohistochemically stained. The morphologies of Myosin7a+ HCs and Sox2+ SCs are shown. *c* Two-way ANOVA followed by Sidak's multiple comparison test was performed and showed that there was no significant difference in the numbers of IHCs and OHCs in *MST1/2*-LoxP mice compared with the control group. *d* Auditory brainstem response (ABR) results showed no significant difference in hearing threshold in *MST1/2*-LoxP mice compared with the normal group analyzed by two-way ANOVA followed by Sidak's multiple comparison test. *$p < 0.05$, **$p < 0.01$, ***$p < 0.001$, ****$p < 0.0001$. Data are shown as the mean ± SEM. Scale bars = 20 μm.

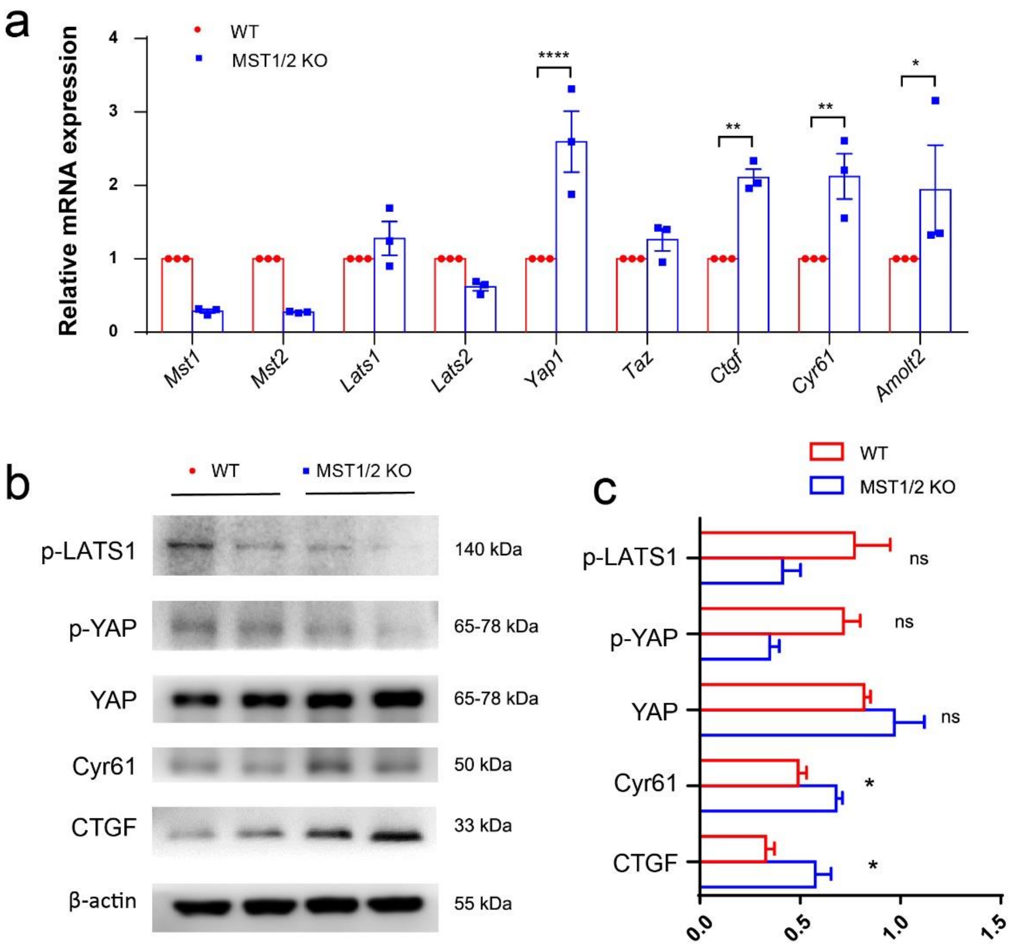

Supplementary figure 6: MST1/2 knockout in vivo reduced endogenous LATS1 and YAP phosphorylation and affected the expression of YAP downstream genes. *a* $Mst1^{fl/fl}/Mst2^{fl/fl}$; Sox9-$CreERT^{2}$/+ mice were used to knock out *Mst1/2* in Sox9+ SCs and thus turn off Hippo signaling in order to promote YAP nuclear translocation. Tamoxifen was injected at P1–2, and the cochleae were harvested at P7. Relative mRNA expression levels of Hippo signaling and downstream genes between $Mst1^{fl/fl}/Mst2^{fl/fl}$; Sox9-$CreERT^{2}$/+ groups and controls are shown, and two-way ANOVA followed by Sidak's multiple comparison test was performed to show the significant differences. *b* Western blotting analysis was performed on cochlear extracts in control and $Mst1^{fl/fl}/Mst2^{fl/fl}$; Sox9-$CreERT^{2}$/+ mice using antibodies against p-LATS, p-YAP, YAP, CRY61, CTGF, and beta-actin. *c* Analysis performed with two-way ANOVA followed by Sidak's multiple comparison test showed that compared with the control group the protein expression levels of p-YAP and p-LATS in the MST1/2 knockout group were decreased and the protein expression levels of CYR61 and CTGF were increased. $*p < 0.05$, $**p < 0.01$, $***p < 0.001$, $****p < 0.0001$. Data are shown as the mean ± SEM.

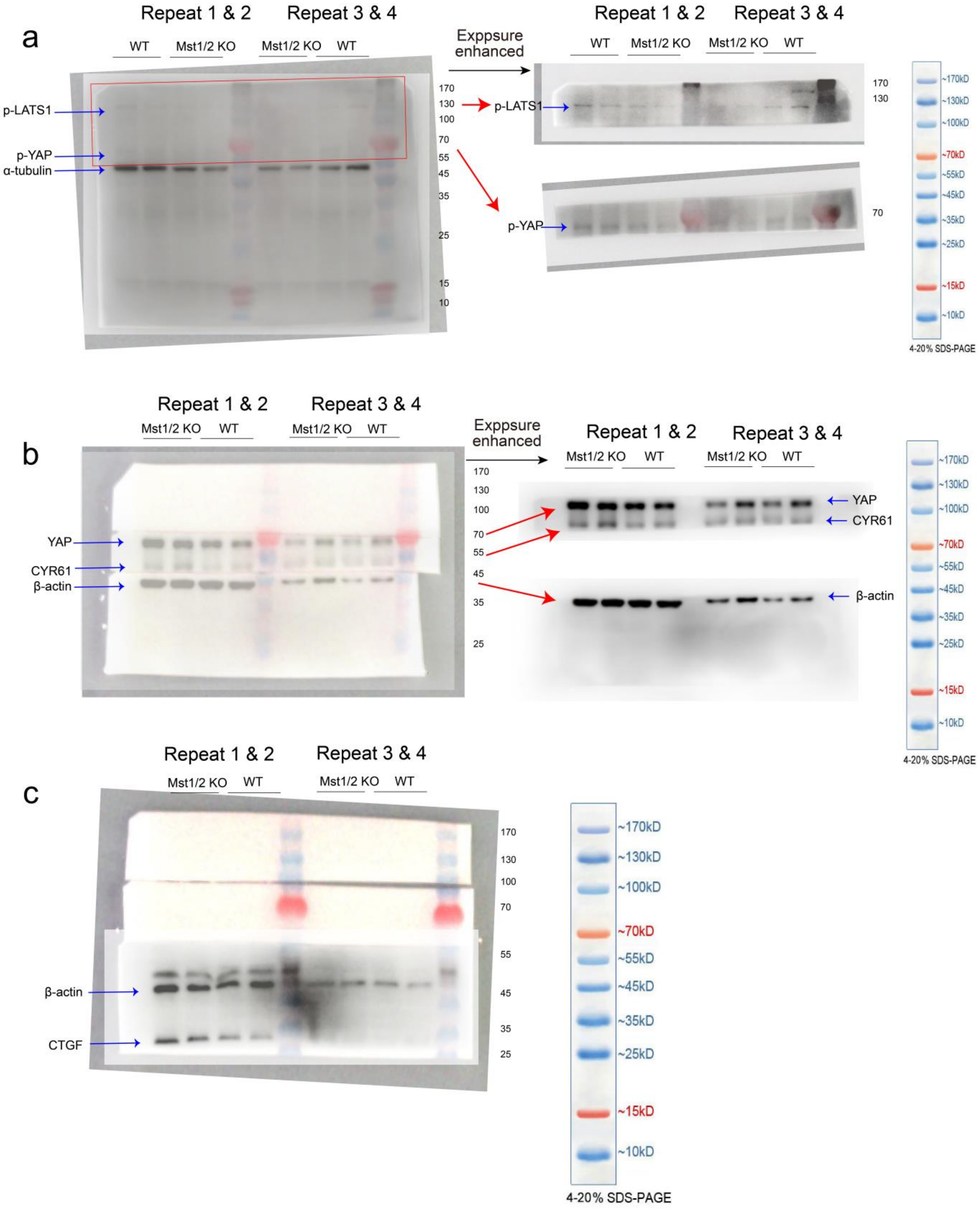

Supplementary figure 7: Unprocessed blots in Supplementary Figure 6*b* used for statistical analysis. *a* Western blotting analysis was performed on extracts of WT and *Mst1/2* KO cochleae using p-LATS1, p-YAP, and α-tubulin. *b* Western blotting analysis was performed on extracts of WT and *Mst1/2* KO cochleae using YAP, CYR61 and β-actin. *c* Western blotting analysis was performed on extracts of WT and *Mst1/2* KO cochleae using CTGF, and β-actin. Chromatograms of the molecular weight standard for color pre-dyed proteins (10-170 kDa).

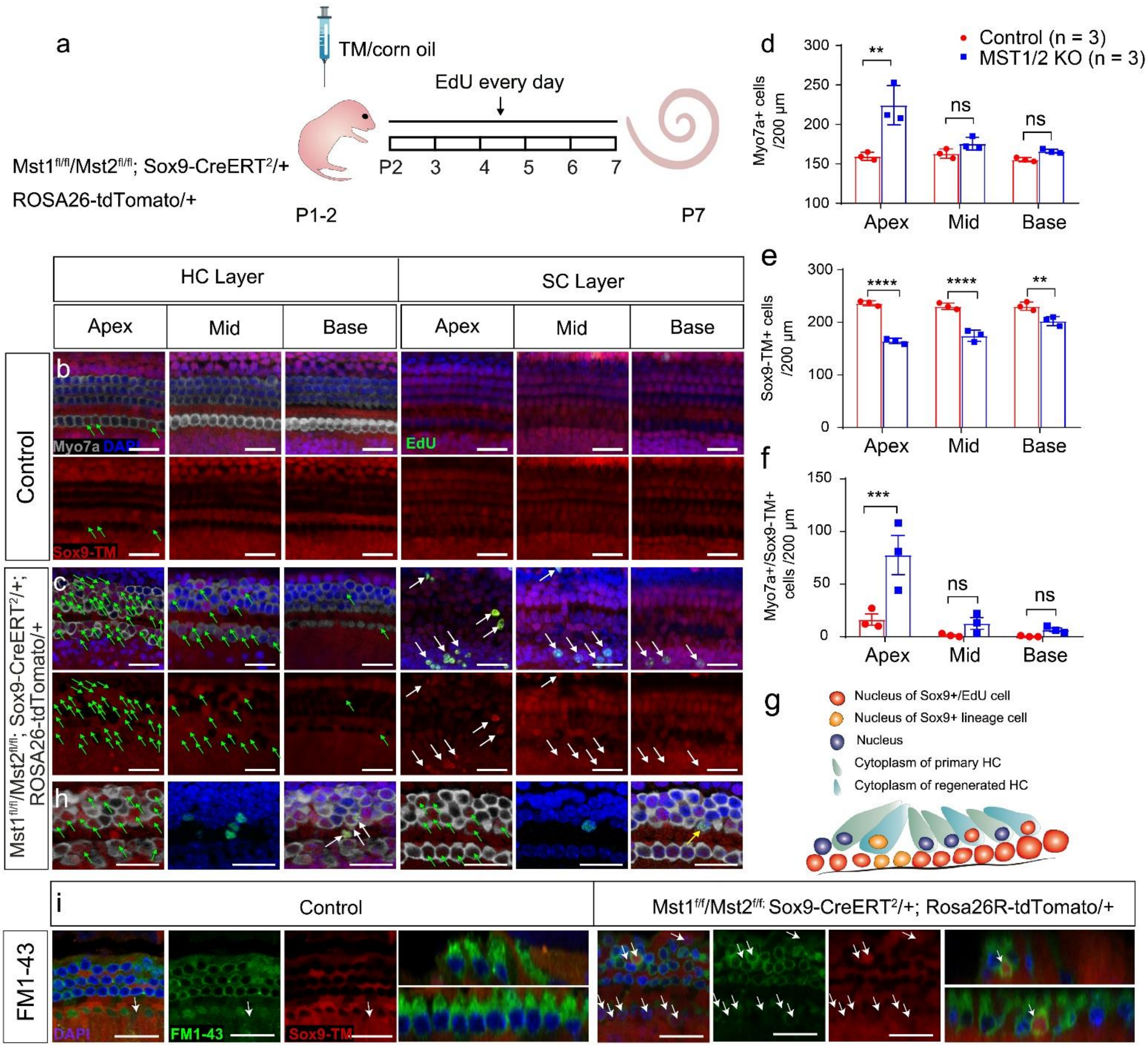

Supplementary figure 8: Regenerated HCs induced by YAP nuclear translocation are derived from the Sox9 lineage *in vivo*. *a* Mst1$^{f1/f1}$/Mst2$^{fl/fl}$; Sox9-CreERT^{2}/+; ROSA26-tdTomato/+ mice with tamoxifen administration at P1–2 were used to lineage trace Sox9+ supporting cells while turning off Hippo signaling. EdU was injected at P2 to P7 to detect proliferating cells. *b* In P7 control mice, Sox9-tdTomato+ cells were almost all SCs in the organ of Corti, and very few HCs (fewer than 5 per cochlea) were Sox9-tdTomato+ (n = 6). *c* There were large numbers of Sox9-tdTomato+ HCs in both IHCs and OHCs in P7 Mst1$^{f1/f1}$/Mst2$^{fl/fl}$; Sox9-CreERT^{2}/+; ROSA26-tdTomato/+ mice. *d-f* Two-way ANOVA followed by Sidak's multiple comparison test was performed and showed that compared with control groups the numbers of HCs were significantly increased and the numbers of Sox9+ SCs were significantly decreased in the SR, and large numbers of newly generated HCs were Sox9+ in the Mst1f1/f1/Mst2fl/fl; Sox9-CreERT2/+ mice. *g* The schematic diagram of the lineage tracing of regenerated HCs. *h* Compared with the number of Sox9-tdTomato+ HCs, very few Sox9-

tdTomato+/EdU+ HCs were observed in P7 $Mst1^{fl/fl}/Mst2^{fl/fl}$; Sox9-$CreERT^2$/+; ROSA26-tdTomato/+ mice, about 0–2 per cochlea. *i* Regenerated HC-like cells *in vivo* were able to uptake FM1-43FX at P7 after knockout of MST1/2 in SOX9+ SCs at P2. $^*p < 0.05$, $^{**}p < 0.01$, $^{***}p < 0.001$, $^{****}p < 0.0001$. Data are shown as the mean ± SEM. Scale bars = 20 μm.

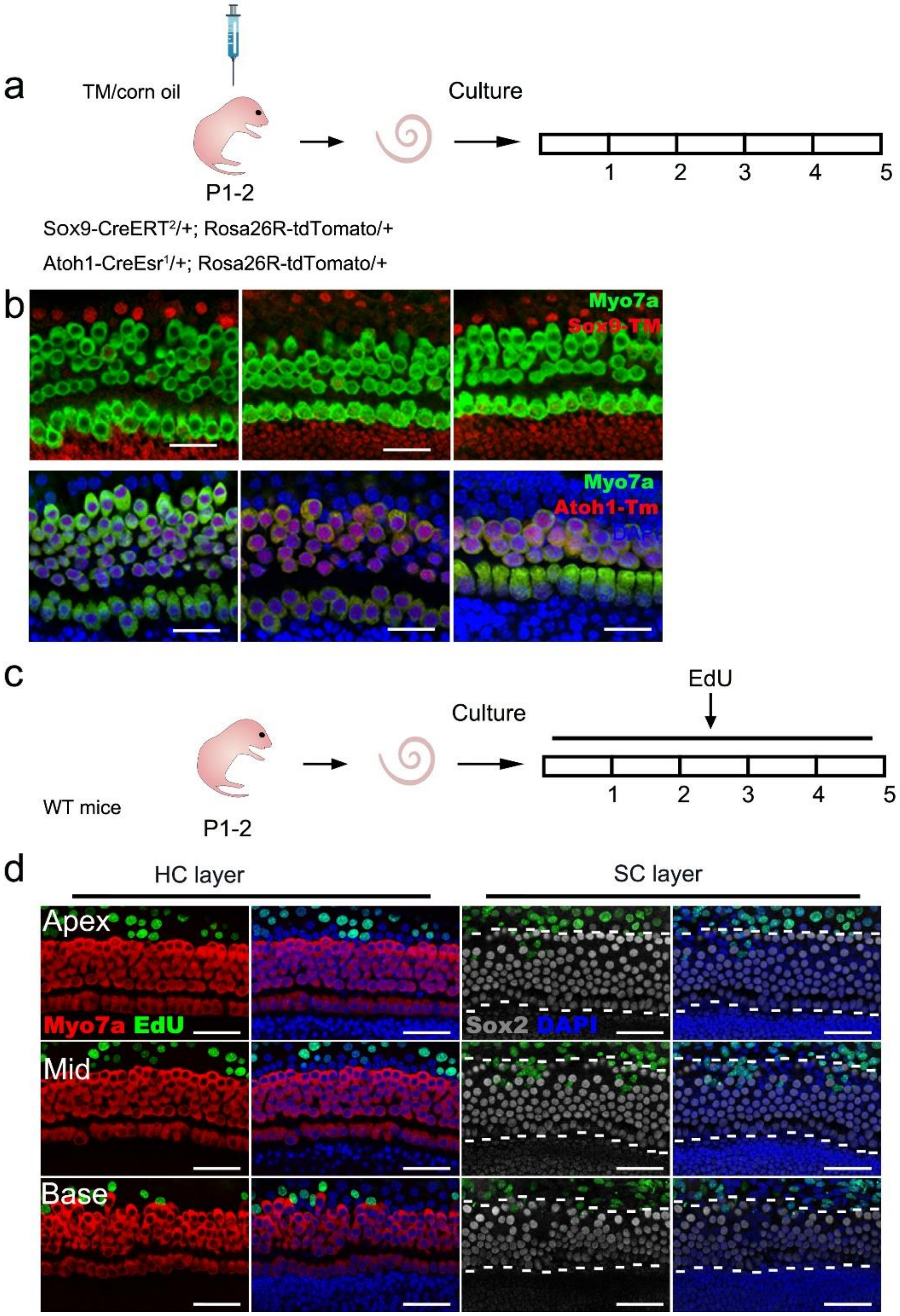

Supplementary figure 9: The cochlear phenotypes of controls without injury. *a* Sox9-CreERT2/+; Rosa26R-tdTomato/+ and Atoh1-CreEsr1/+; Rosa26R-tdTomato/+ mouse models were used to trace the SC and HC lineages. Tamoxifen was injected at P1 to activate tdTomato expression overnight, and the cochleae were harvested at P2 and then cultured in vitro for 5 days. *b* The distribution of Sox9-tdTomato+ cells and Atoh1-tdTomato+ cells from the apex to the base in the HC layer. *c* The cochlear explants from P2 WT mice were harvested and then cultured for 5 days, and 10 µM EdU was added the whole time to trace the proliferating cells. *d* No EdU+ HCs and only rare EdU+ SCs in the SR were observed. Scale bars = 20 µm.

Sup Table1: Organisms/Strains

Mouse: $Mst1^{fl}$ and $Mst2^{fl}$	Boan Li' laboratory	Cat. #017635
Mouse: Rosa26R-tdTomato	Jackson Laboratory	Cat. #7908
Mouse: Sox9-$CreERT^2$	Jackson Laboratory	Cat. #035092
Mouse: Atoh1-Cre/Esr^1	Jackson Laboratory	Cat.#007684

Sup Table2: Genotyping primers

CreER transgene forward	GCG GTC TGG CAG TAA AAA CTA TC
CreER transgene reverse	GTG AAA CAG CAT TGC TGT CAC TT
CreER internal forward	CTA GGC CAC AGA ATT GAA AGA TCT
CreER internal reverse	GTA GGT GGA AAT TCT AGC ATC ATC C
tomato WT forward	AAG GGA GCT GCA GTG GAG TA
tomato WT reverse	CCG AAA ATC TGT GGG AAG TC
tomato mutant reverse	GGC ATT AAA GCA GCG TAT CC
tomato mutant forward	CTG TTC CTG TAC GGC ATG G
Mst1-F	AGTGTTGGCTCTTGATTTTCCT
Mst1-R	CAGGGCTAGAGTGAAACCTTG
Mst2-F	CACACACACACACGGTCTCA
Mst2-R	CCTCCCAGCCTTCCTCTAGT

Sup Table3: Primer pairs for qPCR

Mst1	Forward Primer	CTCACCACTGAATGACTTCCAG
	Reverse Primer	AAGGCCCGACAGTCCAGAA
Mst2	Forward Primer	CGGAGTTACGTGAAAGTTGGT
	Reverse Primer	CAGAAAGAAGGGCCGAATGGA
Lats1	Forward Primer	AAAGCCAGAAGGGTACAGACA
	Reverse Primer	CCTCAGGGATTCTCGGATCTC
Lats2	Forward Primer	GGACCCCAGGAATGAGCAG
	Reverse Primer	CCCTCGTAGTTTGCACCACC
Yap	Forward Primer	TACTGATGCAGGTACTGCGG
	Reverse Primer	TCAGGGATCTCAAAGGAGGAC
Taz	Forward Primer	CCCCCGCTTTGGACAGAAAAT
	Reverse Primer	AGGCTGGAAATGATTGTGGAG
Psx1	Forward Primer	CTGGCTCAACTGCGGTACAG
	Reverse Primer	ACCAATTCTGCACATCACATTCA
Ctnnb1	Forward Primer	ATGGAGCCGGACAGAAAAGC
	Reverse Primer	CTTGCCACTCAGGGAAGGA
Wnt3a	Forward Primer	CTCCTCTCGGATACCTCTTAGTG
	Reverse Primer	GCATGATCTCCACGTAGTTCCTG
Brn3.1	Forward Primer	ATGCGCCGAGTTTGTCTCC
	Reverse Primer	GGGCTTGAACGGATGGTTCT
Axin2	Forward Primer	TGACTCTCCTTCCAGATCCCA
	Reverse Primer	TGCCCACACTAGGCTGACA

Sox2	Forward Primer	GCGGAGTGGAAACTTTTGTCC
	Reverse Primer	CGGGAAGCGTGTACTTATCCTT
Hes1	Forward Primer	CCAGCCAGTGTCAACACGA
	Reverse Primer	AATGCCGGGAGCTATCTTTCT
Hes5	Forward Primer	AGTCCCAAGGAGAAAAACCGA
	Reverse Primer	GCTGTGTTTCAGGTAGCTGAC
Jag1	Forward Primer	CCTCGGGTCAGTTTGAGCTG
	Reverse Primer	CCTTGAGGCACACTTTGAAGTA
Notch1	Forward Primer	CCCTTGCTCTGCCTAACGC
	Reverse Primer	GGAGTCCTGGCATCGTTGG
Cyr61	Forward Primer	CTGCGCTAAACAACTCAACGA
	Reverse Primer	GCAGATCCCTTTCAGAGCGG
Ctgf	Forward Primer	GGGCCTCTTCTGCGATTTC
	Reverse Primer	ATCCAGGCAAGTGCATTGGTA
Actg1	Forward Primer	AATCGCCGCACTCGTCATT
	Reverse Primer	CCCTACGATGGAAGGGAACAC
Amotl2	Forward Primer	GGAGAAGAGTTGCCCACCTAT
	Reverse Primer	TCGAAGAGCTTCATCCTGTCG
Atoh1	Forward Primer	GAGTGGGCTGAGGTAAAAGAGT
	Reverse Primer	GGTCGGTGCTATCCAGGAG

Sup Table4: Antibodies and reagents

**Name**	**Cat. NO**	**Company**	**Source**
DMEM/F12 medium	SH30023.01	Hyclone	GE Healthcare Life Science, Logan, Utah, USA
N2	A1370701	Gibco	Life Technologies, Grand Island, NY, USA
B27	12587010	Gibco	Life Technologies, Grand Island, NY, USA
Ampicillin Sodium Solution	B540722	Sangon Biotech	Sangon Biotech, Shanghai, China
tamoxifen	T5848	Sigma	Sigma-Aldrich, St. Louis, MO, USA
TRIzol	15596018	Ambion	Life Technologies, Carlsbad CA, USA
RIPA	P0013B	Beyotime	Beyotime Biotechnology, China
PMSF	ST506	Beyotime	Beyotime Biotechnology, China
Bicinchoninic acid (BCA) protein kit	P0012S	Beyotime	Beyotime Biotechnology, China
BeyoGel™ Plus Precast PAGE Gel for Tris-Gly System	P0468S	Beyotime	Beyotime Biotechnology, China
Polyvinylidene difluoride	FFP26	Beyotime	Beyotime Biotechnology,

(PVDF) membranes			China
Viagen DirectPCR DNA Extraction System	102-T	Viagen	VIAGEN BIOTECH, Los Angeles, CA, USA
PrimeScriptTM II 1st Strand cDNA Synthesis Kit	6210A	Takara	Takara Bio, Nojihigashi, Kusatsu, Shiga, Japan
TB GreenTM Premix Ex TaqTM II	RR820A	Takara	Takara Bio, Nojihigashi, Kusatsu, Shiga, Japan
Cell-Tak	354241	Corning	Corning, NY, USA
Neomycin	N6386	Sigma	Sigma-Aldrich, St. Louis, MO, USA
XMU-MP-1	S8334	Selleck	Sigma-Aldrich, St. Louis, MO, USA
6-bromoindirubin-3’-oxime	361550	Sigma	Sigma-Aldrich, St. Louis, MO, USA
DAPT	565784	Sigma	Sigma-Aldrich, St. Louis, MO, USA
Rabbit anti-myosin7a	25-6790	Proteus Biosciences	Proteus Biosciences, Ramona, CA, USA
Goat anti-Sox2	AF2018	R&D	R&D System, Minneapolis, MN, USA
Rabbit anti-YAP	14074	CST	Cell Signaling Technology, Danvers, MA, USA
Rabbit anti-Phospho-YAP (Ser127) (D9W2I)	13008S	CST	Cell Signaling Technology, Danvers, MA, USA
Rabbit anti-LATS1(C66B5)	3477S	CST	Cell Signaling Technology, Danvers, MA, USA
Rabbit anti-Phospho-LATS1 (Thr1079) (D57D3)	8654S	CST	Cell Signaling Technology, Danvers, MA, USA
Goat anti-CTGF	SC-14939	Santa Cruz	Santa Cruz Biotechnology, Dallas, USA
Rabbit anti-Cyr61	SC-13100	Santa Cruz	Santa Cruz Biotechnology, Dallas, USA
Mouse anti-Yap1	SC-101199	Santa Cruz	Santa Cruz Biotechnology, Dallas, USA
Alexa Fluor™ 555 Phalloidin	A34055	Invitrogen	Life Technologies Corporation, Eugene, CA, USA
Mouse anti-Tuj1	Ab78078	Abcam	Abcam, Cambridge, UK
FM1-43FX	F35355	Invitrogen	Life Technologies Corporation, Carlsbad, CA, USA
DAPI	D9542	Sigma	Sigma-Aldrich, St. Louis, MO, USA
Click-iT™ EdU Imaging Kit	C10086	Invitrogen	Life Technologies

	Corporation, Carlsbad, CA, USA

Sup table 5: Number of HCs in the organ of Corti from the apex to the base under different treatment conditions (Data are shown as mean ± SEM, *n* = 4).

	Apex	Mid	Bas
Neo	119.500 ± 5.172	68.500 ± 5.737	33.000 ± 3.697
Neo+MP1	163.750 ± 5.360	111.000 ± 6.608	70.250 ± 3.351
Neo+BIO	118.250 ± 11.564	79.250 ± 6.421	33.000 ± 3.916
Neo+MP1+BIO	131.500 ± 10.492	76.000 ± 5.212	20.000 ± 2.915
Neo+DAPT	289.500 ± 17.689	146.250 ± 11.757	82.750 ± 4.553
Neo+MP1+DAPT	354.250 ± 17.556	156.250 ± 15.245	105.000 ± 12.302

Sup table 6: Number of EdU+ SCs in the sensory region in the organ of Corti from the apex to the base under different treatment conditions (Data are shown as mean ± SEM, *n* = 4).

	Apex	Mid	Bas
Neo	1.000 ± 0.408	1.000 ± 0.577	0.000 ± 0.000
Neo+MP1	3.750 ± 0.629	2.500 ± 0.957	2.500 ± 1.258
Neo+BIO	154.500 ± 18.835	47.250 ± 16.665	20.000 ± 6.964
Neo+MP1+BIO	6.000 ± 4.491	0.500 ± 0.500	1.500 ± 1.500
Neo+DAPT	21.500 ± 6.384	5.750 ± 1.315	0.000 ± 0.000
Neo+MP1+DAPT	23.250 ± 8.499	6.500 ± 1.848	0.000 ± 0.000

Sup table 7: Number of EdU+ SCs in the greater epithelial ridge in the organ of Corti from the apex to the base under different treatment conditions (Data are shown as mean ± SEM, *n* = 4).

	Apex	Mid	Bas
Neo	0.250 ± 0.250	0.000 ± 0.000	0.000 ± 0.000
Neo+MP1	7.500 ± 1.041	3.250 ± 0.750	1.000 ± 0.707
Neo+BIO	178.000 ± 26.997	95.500 ± 21.411	0.750 ± 0.750
Neo+MP1+BIO	160.000 ± 14.053	101.000 ± 14.445	0.000 ± 0.000
Neo+DAPT	18.000 ± 6.014	2.750 ± 1.601	0.000 ± 0.000
Neo+MP1+DAPT	215.250 ± 32.227	115.750 ± 43.936	17.250 ± 7.192

Sup table 8: Number of EdU+ HCs in the organ of Corti from the apex to the base under different treatment conditions (Data are shown as mean ± SEM, *n* = 4).

	Apex	Mid	Bas
Neo	0.000 ± 0.000	0.000 ± 0.000	0.000 ± 0.000
Neo+MP1	2.000 ± 0.707	2.250 ± 0.629	2.000 ± 0.816
Neo+BIO	0.000 ± 0.000	0.250 ± 0.250	0.000 ± 0.000
Neo+MP1+BIO	1.500 ± 0.957	1.750 ± 1.181	0.750 ± 0.479
Neo+DAPT	38.000 ± 3.719	2.250 ± 1.109	0.000 ± 0.000
Neo+MP1+DAPT	39.250 ± 5.250	3.500 ± 1.323	0.000 ± 0.000
